# Systematic Analysis of Splice-Site-Creating Mutations in Cancer

**DOI:** 10.1016/j.celrep.2018.03.052

**Published:** 2018-04-03

**Authors:** Reyka G. Jayasinghe, Song Cao, Qingsong Gao, Michael C. Wendl, Nam Sy Vo, Sheila M. Reynolds, Yanyan Zhao, Héctor Climente-González, Shengjie Chai, Fang Wang, Rajees Varghese, Mo Huang, Wen-Wei Liang, Matthew A. Wyczalkowski, Sohini Sengupta, Zhi Li, Samuel H. Payne, David Fenyö, Jeffrey H. Miner, Matthew J. Walter, The Cancer Genome Atlas Research Network, Benjamin Vincent, Eduardo Eyras, Ken Chen, Ilya Shmulevich, Feng Chen, Li Ding

**Affiliations:** 1 Department of Medicine, Washington University in St. Louis, St. Louis, MO 63110, USA; 2 McDonnell Genome Institute, Washington University in St. Louis, St. Louis, MO 63108, USA; 3 Division of Oncology, Washington University in St. Louis, St. Louis, MO 63110, USA; 4 Department of Genetics, Washington University in St. Louis, St. Louis, MO 63110, USA; 5 Department of Mathematics, Washington University in St. Louis, St. Louis, MO 63130, USA; 6 Department of Bioinformatics and Computational Biology, The University of Texas MD Anderson Cancer Center, Houston, TX, USA; 7 Institute for Systems Biology, Seattle, WA 98109, USA; 8 Institut Curie, 75248 Paris Cedex, France; 9 MINES ParisTech, PSL-Research University, CBIO-Centre for Computational Biology, 77300 Fontainebleau, France; 10 INSERM U900, 75248 Paris Cedex, France; 11 Lineberger Comprehensive Cancer Center, The University of North Carolina at Chapel Hill, Chapel Hill, NC, USA; 12 Curriculum in Bioinformatics and Computational Biology, The University of North Carolina at Chapel Hill, Chapel Hill, NC, USA; 13 Division of Nephrology, Washington University in St. Louis, St. Louis, MO 63110, USA; 14 Department of Biochemistry and Molecular Pharmacology, New York University School of Medicine, New York, NY 10016, USA; 15 Institute for Systems Genetics, New York University School of Medicine, New York, NY 10016, USA; 16 Biological Sciences Division, Pacific Northwest National Laboratory, Richland, WA, USA; 17 Siteman Cancer Center, Washington University in St. Louis, St. Louis, MO 63110, USA; 18 Catalan Institution of Research and Advanced Studies (ICREA), 08010 Barcelona, Spain; 19 Computational RNA Biology Group, Pompeu Fabra University (UPF), 08003 Barcelona, Spain

## Abstract

For the past decade, cancer genomic studies have focused on mutations leading to splice-site disruption, overlooking those having splice-creating potential. Here, we applied a bioinformatic tool, MiSplice, for the large-scale discovery of splice-site-creating mutations (SCMs) across 8,656 TCGA tumors. We report 1,964 originally mis-annotated mutations having clear evidence of creating alternative splice junctions. *TP53* and *GATA3* have 26 and 18 SCMs, respectively, and *ATRX* has 5 from lower-grade gliomas. Mutations in 11 genes, including *PARP1*, *BRCA1*, and *BAP1*, were experimentally validated for splice-site-creating function. Notably, we found that neoantigens induced by SCMs are likely several folds more immunogenic compared to missense mutations, exemplified by the recurrent GATA3 SCM. Further, high expression of PD-1 and PD-L1 was observed in tumors with SCMs, suggesting candidates for immune blockade therapy. Our work highlights the importance of integrating DNA and RNA data for understanding the functional and the clinical implications of mutations in human diseases.

## In Brief

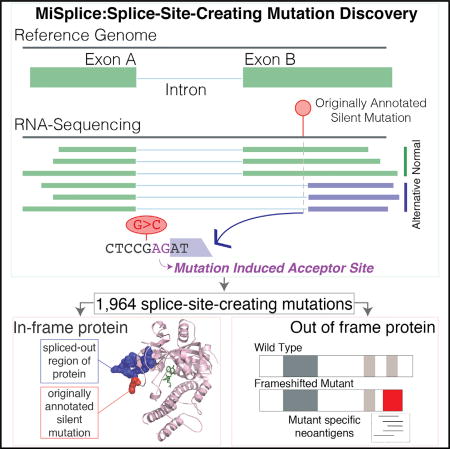

Jayasinghe et al. identify nearly 2,000 splice-site-creating mutations (SCMs) from over 8,000 tumor samples across 33 cancer types. They provide a more accurate interpretation of previously misannotated mutations, highlighting the importance of integrating data types to understand the functional and the clinical implications of splicing mutations in human disease.

## INTRODUCTION

Large-scale sequencing studies, such as The Cancer Genome Atlas (TCGA) project, have worked to address the functional consequences of genomic mutations in tumors (Dees et al., 2012; Kandoth et al., 2013; Lawrence et al., 2013; Niu et al., 2016), with the larger goal of determining the underlying mechanisms of cancer initiation and progression. Many studies have focused on characterizing (1) non-synonymous somatic mutations that alter amino acid sequence and (2) splice-disrupting mutations at splice donors and acceptors (Jung et al., 2015). Current annotation methods typically classify mutations as disruptors of splicing if they fall on either the consensus intronic dinucleotide splice donor, GT, or the splice acceptor, AG. As a group, splice site mutations have been presumed to be invariably deleterious because of their disruption of the conserved sequences that are used to identify exon-intron boundaries.

While this classification method has been useful, increasing evidence suggests that splice site mutations can lead to transcriptional changes beyond disruption of the canonical junction (Lim and Fairbrother, 2012; Mort et al., 2014; Rivas et al., 2015; Sauna and Kimchi-Sarfaty, 2011; Steffensen et al., 2014). One such example is the c.190 mutation in *BRCA1*. Conventional annotation had predicted a missense mutation, p.C64G, but our analysis of RNA sequencing (RNA-seq) data in ovarian tumors harboring p.C64G and a published mouse model (Yang et al., 2003) suggested the germline c.190 mutation leads to the creation of an alternative splice junction, resulting in a truncated null protein. Several case studies have reported observations of missense and silent mutations activating cryptic splice sites in *MLH1* (Nyström-Lahti et al., 1999), *LMNA* (Woolfe et al., 2010), *RB1* (Zhang et al., 2008), *RNASEH2A* (Rice et al., 2013), *MECP2* (Sheikh et al., 2013), *BAP1* (Wadt et al., 2012), and *KIT* (Chen et al., 2005), and other studies relate missense and silent mutations to splicing changes (Jung et al., 2015; Kahles et al., 2016; Soemedi et al., 2017; Supek et al., 2014). Despite the broad clinical ramifications of mutation-induced altered splicing, a systematic evaluation of their occurrence and the resultant effects in cancer has yet to be undertaken, and there have not been significant bioinformatics platforms for doing so.

We developed a bioinformatic tool called MiSplice (mutation-induced splicing) that integrates DNA and RNA-seq data across thousands of samples to discover mutations that induce splice site creation. In our large-scale analysis across 8,656 tumor samples, we report 1,964 such somatic mutations that had originally been mis-annotated. Splice-site-creating mutations (SCMs) are enriched in the new introns, with the highest rate at the −3 nt position of acceptors with two-thirds of such events at aGag and agGag repeats by creating an alternative junction 2 nt away. Partial and full splice creation capabilities across these 1,964 sites were evaluated by measuring the fraction of reads supporting the alternative junction, which we termed the ‘‘junction allele fraction’’ (JAF) and which is found to be negatively correlated with distance to the new splice site. In total, 1,607 genes harbor SCMs, with 248 of them having more than one mutation, including *TP53, GATA3, ATRX*, and *NF1*. Recurrent SCMs were found in *TP53*, *GATA3*, *DDX5*, *KDM6A*, *PTEN*, *SETD2*, *SMAD4*, *BCOR*, *SPOP*, and *BAP1*, suggesting an association with cancer development. Broadly speaking, integrated DNA and RNA data can furnish a sound basis for discovering SCMs and for accurately understanding functional consequences of mutations in cancer and in other human diseases.

## RESULTS

### Splice-Site-Creating Mutation Discovery

**Figure 1:**
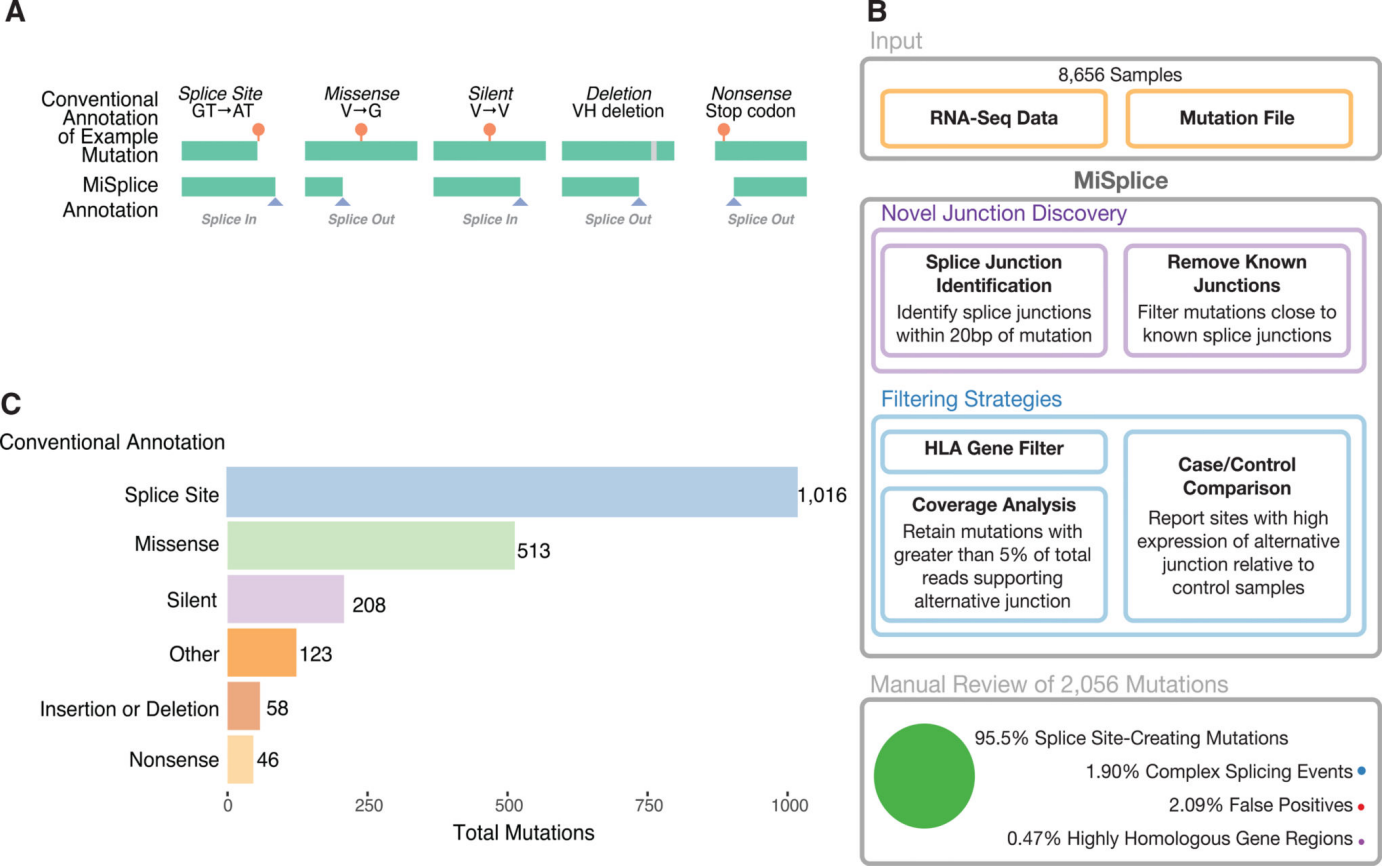
Splice-Site-Creating Mutation Discovery (A) Examples of splice-site-creating mutations for different conventionally annotated mutation types. Splice-in is defined as mutations contained within the newly created exons, and splice-out is when the mutation is present in the newly created intron. (B) The MiSplice workflow consists of three steps: alternative junction discovery, filtering, and manual review. First, the user inputs the locations of RNA-seq BAM files along with a mutation file. MiSplice searches the BAM file to identify any alternative splice junctions near the mutation of interest, while filtering out known splice junctions and calculating the number of alternative junction-supporting reads for case and control samples. For the filtering step, the following sites are removed: mutations in HLA genes, a low fraction of reads supporting the alternative splice junction, and sites expressed in controls. Finally, we manually reviewed all sites to validate the *in silico* predictions. (C) Breakdown of 2,056 manually validated splice-site-creating mutations by conventional annotation.

We collected high-quality mutation calls from 8,656 tumors across 33 cancer types derived from The Cancer Genome Atlas having available TCGA RNA-seq data (STAR Methods). For every mutation, we defined a set of control samples in the same cancer cohort that lacked the same mutation in the gene of interest. We sought to assess the landscape of SCMs across cancer genomes by evaluating all mutations already having conventional annotations and their potential splice-site-creating effects (Figure 1A). To achieve this goal, we conducted analysis using a bioinformatic tool, MiSplice (mutation-induced splicing), that systematically evaluates mutations in a splicing context using RNA-seq data (Figure 1B).

MiSplice manages large analyses using parallel computation to search for alternative splice junctions within windows of ±20 bp from the mutation of interest. For example, of the 1,416,566 candidate mutations examined here, 4,448 had five or more unique RNA-seq reads supporting the predicted alternative junction in proximity to the mutation. MiSplice then conducts a series of further evaluations, including Ensembl-based filtering of canonical junctions, establishing observational significance by case comparison to a matched set of controls, and assessing score and depth of each cryptic site using MaxEntScan (Yeo and Burge, 2004) and SamTools (Li et al., 2009). From the resultant subset, MiSplice filters out human leukocyte antigen (HLA) genes and sites whose junctions have insufficient difference of expression, as judged from the case-control assessment. Here, we evaluated promising alternative junctions with at least 5% of paired-end RNA-seq reads at the genomic location supporting the alternative junction of interest.

MiSplice processing revealed 2,056 mutations (Table S1) that potentially create an alternative splice site. Manual review indicated a 2.09% false-positive rate, suggesting high specificity of the MiSplice algorithm for discovering these types of mutation-induced splicing events. Of these putative splice events, 1.90% and 0.47% are considered complex and are in highly homologous gene regions, respectively, so they were excluded from further analyses (STAR Methods).

Of the final 1,964 SCMs passing manual review (Table S1), 52% (1,016) are in annotated splice sites, suggesting disruption of the canonical splice site and selection of a the alternative splice site nearby (Figure 1C). Importantly, 26% (513) and 11% (208) of the SCMs had previously been mis-annotated as missense and silent mutations, respectively. In addition, we found 58 insertions or deletions, 46 nonsense, and 123 non-coding region mutations that likewise create cryptic splicing sites.

### Molecular and Biological Patterns of SCMs

Next, we characterized the sequence context for the 1,790 SCMs corresponding to single nucleotide mutations. The sequences of each 9-mer (donor) and 23-mer (acceptor) covering the mutation position were extracted for both the mutant and the reference sequences. Their splice scores as potential donor or acceptor sites were then estimated using MaxEntScan (Table S1).

**Figure 2:**
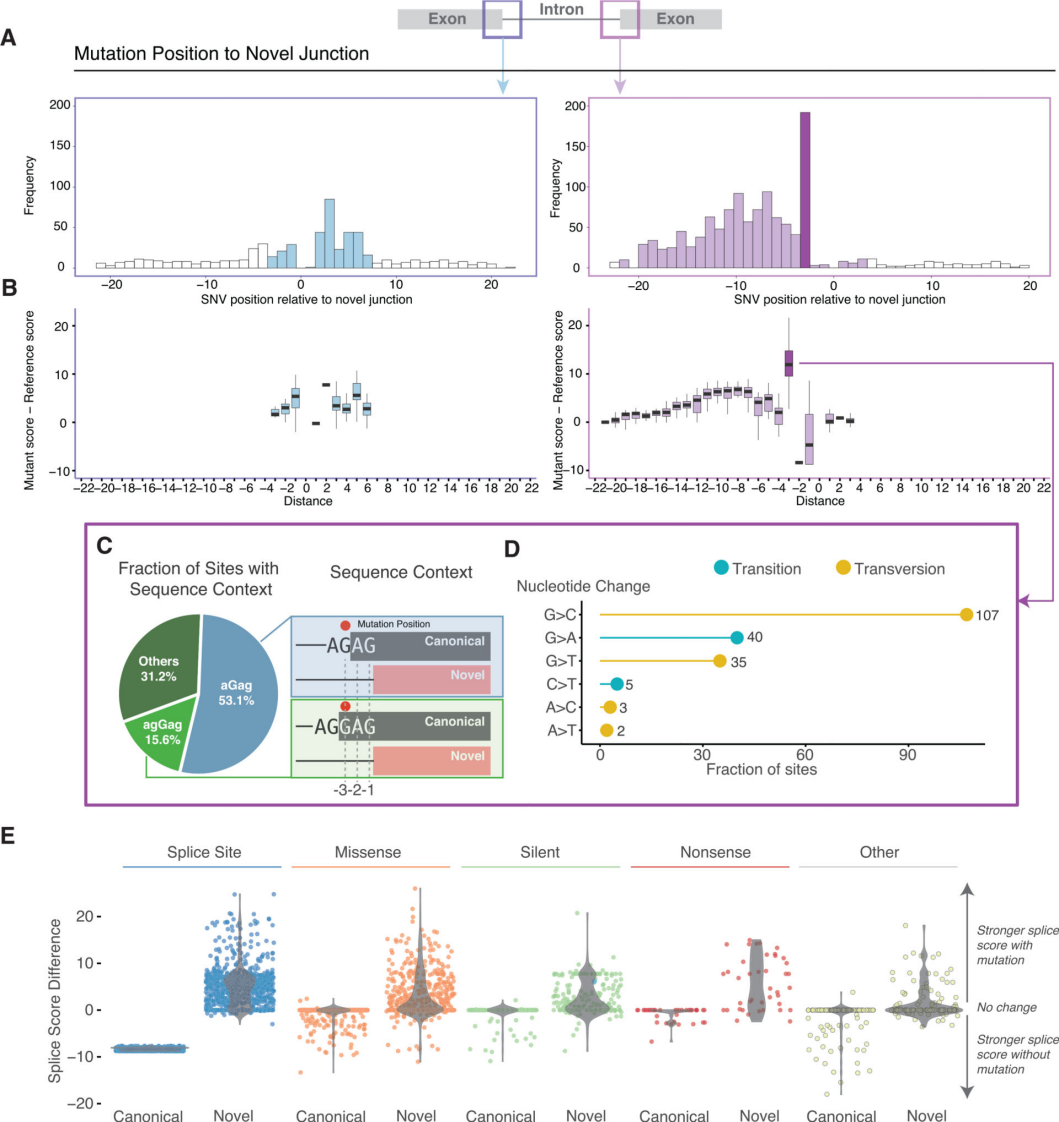
Sequence Contexts and Characteristics of Splice-Site-Creating Mutations (A) Frequency distribution of splice-site-creating mutations relative to the newly created splice junction, with high frequency shown at the third nucleotide position in the newly created intron. (B) Comparison of splicing scores for the newly created splice site, before (reference) and after the mutation (mutant). A larger effect of mutations at the third nucleotide position in the intron (especially for the 3′ splice sites) is shown. (C) Dominant nucleotide sequence context for splice-site-creating mutations at −3 position of the 3′ splice site. Mutation position (red dot) is present 3 base pairs away from the newly created exon. (D) Transition and transversion rate at the −3 position of the 3′ splice site. Most mutations are G > C transversions, strengthening the consensus sequence of the splicing factor U2AF1. (E) Comparison of splicing scores between the nearest canonical splice junction with and without a mutation compared to the newly created splice junction with and without a mutation. Most mutations strengthen the alternative splice junction relative to the canonical splice junction.

Mutations near the alternative splice junctions show higher mutation rates in the introns for both 5′ (p < 1 × 10^−5^, binomial test) and 3′ splice site (p < 1 × 10^−6^) (Figure 2A). More interestingly, we found an enrichment of mutations at the third nucleotide position in the intron, but depletion at the first and second positions (especially for 3′ splice site) (Figure 2A). Comparison of splicing scores between splice-site-creating mutants and reference forms shows that most mutants have stronger splice signals than the reference (Figure 2B). Mutations that create a G or T to produce an alternative 5′ splice site dramatically increase splice site strength. For 3′ splice sites, mutations enriched on the third nucleotide of the newly created intron showed the largest increase of splicing score (Figure 2B). Further examination of the sequence context around mutations at the third nucleotide of 3′ splice sites shows that 53% have a mutation on aGag repeats and another 16% are mutated on agGag repeats, all creating alternative junctions 2 nt away from the annotated ones (Figure 2C). Mutations at the −3 position of the alternative acceptor site would potentially enhance *U2AF1* recognition of the acceptor splice site. Previous studies have reported S34F *U2AF1* mutants preferentially skip exons that contain a T nucleotide at the −3 position (Okeyo-Owuor et al., 2015). Of the 192 mutations located at the −3 position from the alternative junction and that contain an AG in the −2 and −1 positions, 56% undergo a G > C transversion (21%G > A, 18%G > T,3%C > T,2%A > C, 1% A > T), with C being the preferred base at the −3 position for *U2AF1* binding (Figure 2D).

We also explored the relationship between the alternative and canonical splice junctions. As expected, mutations at splice sites dramatically reduced splice scores of the canonical splice junctions, while strengthening those at the alternative splice junctions in most cases. In contrast, a subset of missense and silent mutations did not drastically alter the canonical junction, but instead preferentially strengthened a nearby alternative splice site (Figure 2E). When analyzing the raw splicing scores (canonical and alternative site before and after mutation), we found that 1,089 out of 1,790 (61%) events showed higher splice score for the alternative splice site than the canonical site, indicating inclination for the alternative sites. Further, while 485 (27%) events saw lower post-mutation alternative splice score, differences between alternative and canonical scores had decreased, suggesting that these mutations are still likely enhancing the preference for the alternative site. Only 214 (12%) events did not show evidence, suggesting increased post-mutational preference for using the alternative site. These cases are a good illustration of the fact that many other genomic splicing features are also relevant, including exonic splicing enhancers (ESE), polypyrimidine tract, branch point, and RNA-binding proteins. They are also consistent with the general view that splice score is not definitive (Jian et al., 2014). We emphasize that all 1,790 alternative splice sites demonstrated usage based on patient RNA-seq data and that 10 out of 11 (>90%) identified SCMs were validated experimentally (see below).

### Expressivity and Penetrance of SCMs

**Figure 3:**
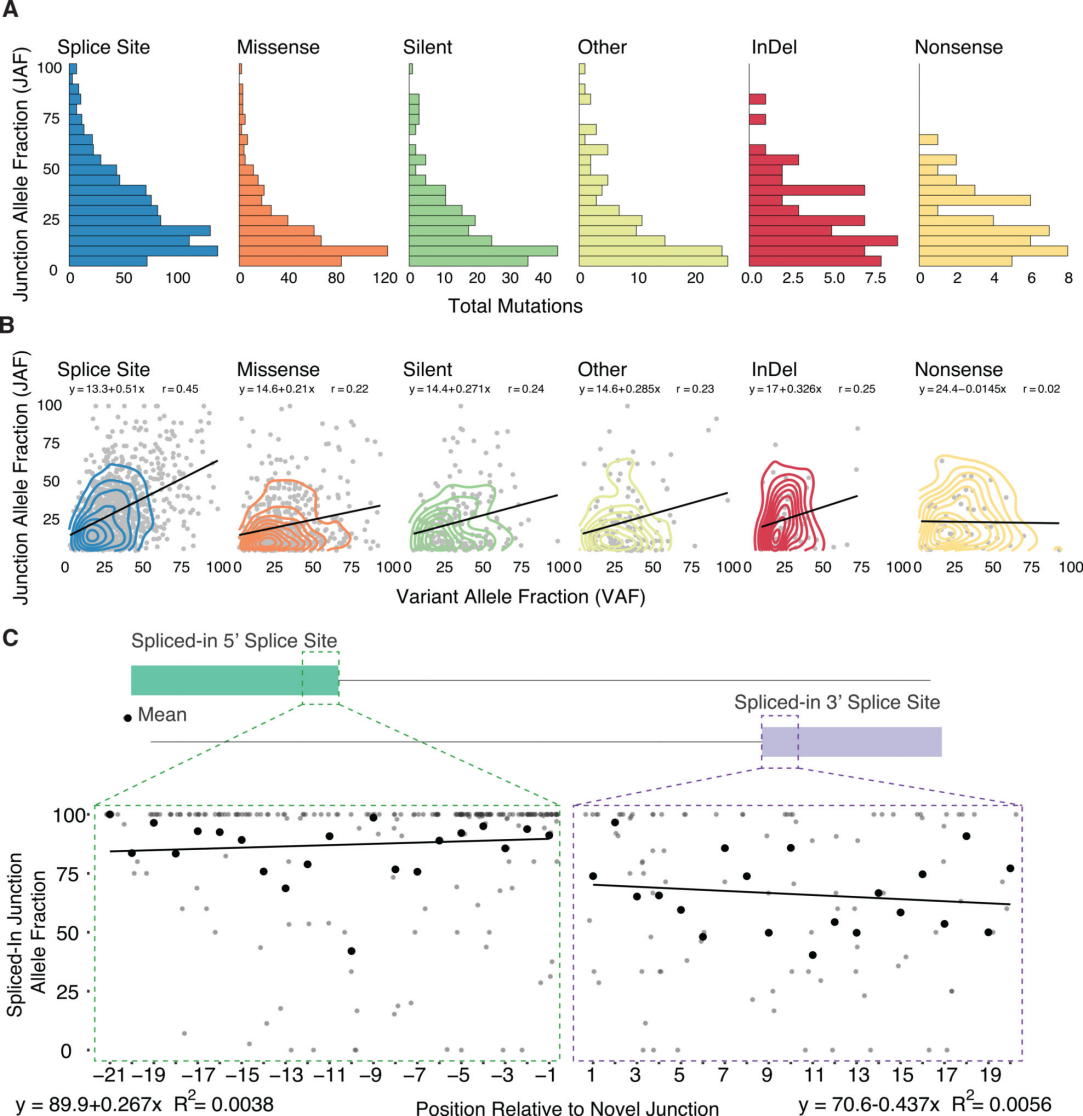
Junction Allele Fraction of Splice-Site-Creating Mutations (A) The junction allele fraction (JAF) is defined as the number of reads supporting the alternative spliced junction relative to total junction spanning reads. Distribution of JAF values separated by conventional annotation type. (B) JAF versus DNA variant allele fraction (VAF) comparison by conventional annotation type. Most mutation types show a generally positive correlation between JAF and VAF values. (C) Splice-site-creating mutations expressed in the newly created exon of the alternative splice junction. Comparison of mutation position relative to the percent of reads supporting the alternative junction and mutation (spliced-in JAF). The mean of each position is highlighted by the black point. For all positions, there is a strong correlation between the presence of the splice-site-creating mutation and the alternative splice junction.

In the presence of the mutation, alternative splice junctions exhibited a wide range of expression. To quantify this effect, we measured alternative junction expression as the fraction of alternatively spliced junction spanning reads over the total number of reads at the genomic location, what we refer to as the JAF. Figure 3A shows the distribution of JAF’s for all high confidence MiSplice predicted alternative junctions, separated by conventional mutation annotations (Figure 3A). Currently, we use a JAF cutoff of 5% for reporting the final high-confidence sites. However, there are some potential alternative sites excluded by this cutoff. Our analysis revealed alternative junction expression varies widely. As expected, DNA variant allele fraction (VAF) and JAF have a generally positive correlation (Figure 3B), with SCMs in *KDM6A* and *FGFR2* having >75% DNA VAF and JAF. However, a SCM in *ARID1A* has a DNA VAF of 23% and JAF of 67%. Such large ranges have been noted for mutations outside of the splice site (Broeks et al., 2003; Clarke et al., 2000; Venables, 2004). Both the truncated and normal spliced products can be observed for many variants, due to either the wild-type allele or leaky splicing, for example, as observed in *RNASEH2A, NFU1, SMN1, CFTR*, and *NF2* (Boerkoel et al., 1995; Caminsky et al., 2014; Ferrer-Cortès et al., 2016; Lohmann and Gallie, 2004; Mautner et al., 1996; Pagani et al., 2003; Rice et al., 2013; Svenson et al., 2001; Vezain et al., 2011).

Next, we considered the expression of mutations that are spliced-in, i.e., mutations located within the exon of the alternatively spliced product. To this end, we determined the ratio of the number of alternative junction reads containing the mutation versus total number of reads supporting the alternative junction (Figure 3C; Table S1). Overall, most of the reads supporting the alternative junction also support the mutation, a finding that suggests a strong association between the mutation and alternative splice junction. Regarding the 5′ splice site, mutations within the first 6 bp of the new exon junction have a much higher fraction of alternative junction reads supporting them; and we see an inverse correlation between the mutation and the junction as the distance between them increases. For the 3′ splice site, we observe a similar trend, although with a higher variability as a function of the distance from the alternative junction.

### SCMs across Genes and Cancer Types

**Figure 4:**
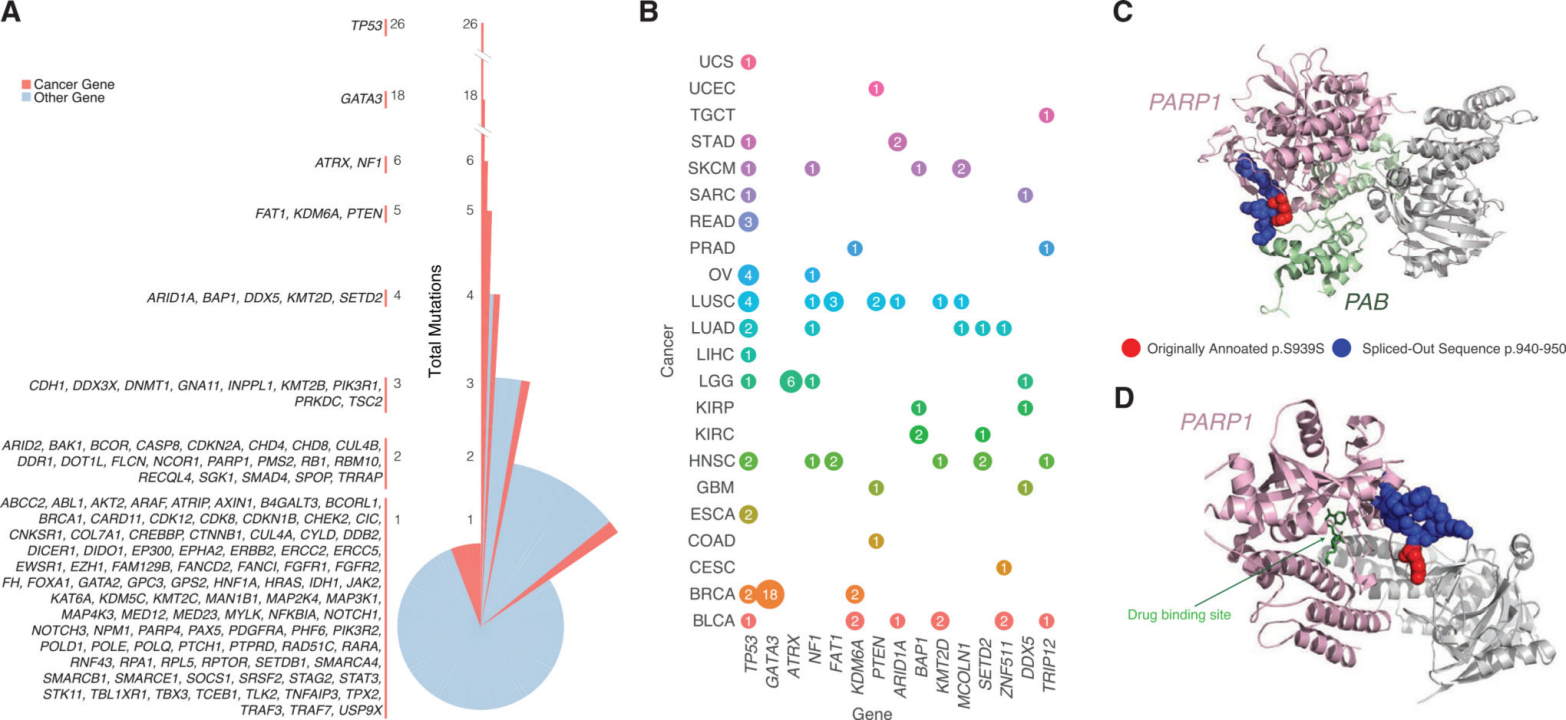
Splice-Site-Creating Mutations across Genes and Cancer Types (A) Distribution of splice-site-creating mutations in each gene separated by the total number of mutations in each gene. *TP53* has the largest number of splice-site-creating mutations, followed by *GATA3* and *ATRX*. (B) Genes with the highest number of pancancer splice-site-creating mutations. Circle size correlates with the total number of mutations for each gene (labeled inside circle) and colored by cancer type. Splice-site-creating mutations in *TP53* are present in many cancer types, while mutations in *ATRX* and *GATA3* are specific to LGG and BRCA, respectively. (C) Proteins Timeless (PAB domain) and PARP1 (chain A) are colored green and pink, respectively. Originally annotated p.S939S mutation (red) and spliced-out sequence (blue) are highlighted on PARP1 (chain A). (D) 3D protein structure of PARP1 in complex with an inhibitor (PDB ID: 5WRQ). Drug inhibitor and PARP1 (chain A) are indicated in green and pink, respectively.

A total of 1,607 unique genes harbored SCMs, with 85% (1,359) having one mutation and 15% (248) having two or more. *TP53* contained the greatest number (26), followed by *GATA3* (18). While most SCMs were found outside the current cancer gene compendium (Table S1), Figure 4A shows that a remarkable number of cancer genes harbor splice altering variants, a phenomenon supported in the literature (Sebestyén et al., 2016). A pan-cancer view reveals that *TP53* was the most mutated across cancer types, while 18 *GATA3* mutations and 6 *ATRX* mutations were specific to breast cancer (BRCA) and lower-grade glioma (LGG), respectively.

We observed 137 mutations nearby to one another (±5 bp) which lead to the creation of the same recurrent splice-site-creating events, not only in *TP53* but also in *GATA3*, *DDX5*, *KDM6A*, *SETD2*, *PTEN, SPOP*, and *BAP1*. While some mutations did not occur at the same position, 14 mutations creating the same alternative splice junction were found in the same exon, including 2 mutations in the third exon of *BAK1*. While most mutations in close proximity created the same alternative splice junction, two adjacent SCMs in *CTNND1* and 2 nearby exonic mutations in *ACP2* and *GMPPB* created different alternative junctions.

**Figure 5:**
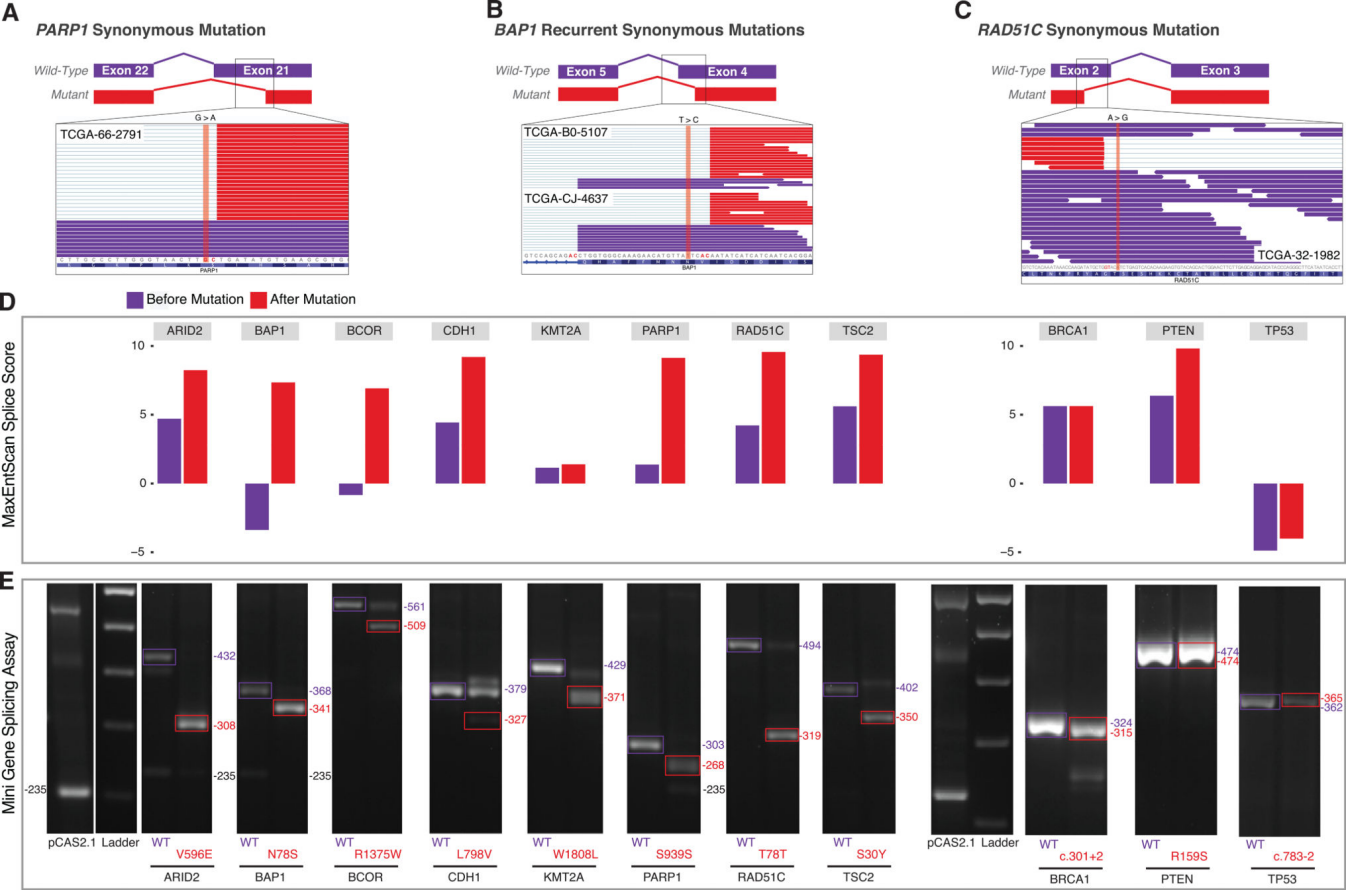
Minigene Functional Assay of Splice-Site-Creating Mutations (A) Integrative genomics viewer (IGV) screenshot of the conventionally annotated synonymous mutation in *PARP1* in exon 21. RNA-seq reads of the candidate splice-site-creating mutation reveal the creation of an alternative splice site (red reads) created by the conventionally annotated synonymous mutation. (B) Candidate recurrent splice-site-creating mutations in *BAP1*. Conventionally annotated as synonymous variants, the *BAP1*-mutated region shows alternatively spliced reads (red reads) in the IGV screenshot for each sample with the splice-site-creating mutation. (C) IGV screenshot of a conventionally annotated synonymous mutation in *RAD51C* in exon 2. (D) Maximum entropy score of the splice-site-creating variant before (purple) and after (red) the introduced mutation for each variant functionally validated in the mini-gene splicing assay. *In silico* predictions suggest all mutations strengthen the alternative splice site. (E) Candidate splice-site-creating mutations validated by the mini-gene splicing assay. Exons of interest were cloned into the pCAS2.1 vector and mutant (red); wild-type (purple) plasmids were transfected into 293T cells; and total RNA was extracted to identify mutation-induced alternatively spliced products.

SCMs can impact protein structure and have potential therapeutic implications. Poly ADP-ribose polymerase 1 (*PARP1*) is an enzyme involved in recruiting protein members of DNA repair pathways including Timeless PAB (PARP1 binding domain) (Figure 4C) (Xie et al., 2015). Since *PARP1* is essential to many cellular processes, including DNA repair, it is commonly targeted by antitumor agents (Malyuchenko et al., 2015). *PARP1* inhibitors targeting the catalytic domain disrupt DNA repair mechanisms thereby increasing the effectiveness of chemotherapeutic agents (Figure 4D). Identifying mutations that disrupt inhibitor binding are essential to properly evaluate treatment options. MiSplice identified a conventionally annotated silent *PARP1* mutation (p.S939S) in a lung squamous cell carcinoma (LUSC) patient that acts as a splice-site-creating variant by creating a *de novo* donor site (Figure 5A). 82 reads supported the *de novo* donor site, which results in a 10 amino acid deletion (p.940–p.950) that falls within the catalytic domain (Figure 4D). Out of 173 LUSC control samples, none contained reads supporting the alternative junction, providing strong evidence that the annotated ‘‘silent’’ mutation is actually a SCM. Previous reports of missense mutations at p.940 are predicted to reduce *PARP1* enzymatic activity by disrupting the binding affinity of *PARP1* to its substrate NAD+ (Alshammari et al., 2014). The in-frame SCM likely disturbs the local structure of *PARP1* and thereby disrupts the interactions between PARP1, its protein binding partners, and drugs binding within the pocket (Figures 4C and 4D).

We identified two kidney renal clear cell carcinoma (KIRC) samples having the same conventionally annotated missense mutation (c.233A > G, p.N78S) in *BAP1*, a nuclear deubiquitinase, that created the same spliced-out alternative splicing product (Figure 5B). Inactivation of *BAP1* is prevalent among renal cell carcinomas (Peña-Llopis et al., 2012) and an annotated missense mutation (p.L570V) has been reported to create a cryptic splice site in melanoma (Wadt et al., 2012). At the transcriptional level, the expressions of the case and control samples are relatively comparable, but at the translational level, one case with available protein data (RPPA) showed significantly lower expression (p = 0.044, permutation test) relative to the controls (Figure S1; Table S2). This result suggests the conventionally annotated missense mutations in *BAP1* likely create an alternatively spliced transcript that is not readily expressed at the protein level.

We used a pCAS2.1 splicing reporter mini-gene functional assay that was adapted from previous publications (Bonnet et al., 2008; Gaildrat et al., 2010; Malone et al., 2016; Tournier et al., 2008; Vreeswijk and van der Klift, 2012), to validate SCMs in 11 cancer genes, including two originally annotated silent mutations in *PARP1*, *RAD51C*, two splice site mutations in *TP53* and *BRCA1*, and several missense mutations in *ARID2*, *BAP1*, *BCOR*, *CDH1*, *KMT2A, PTEN*, and *TSC2*. Wild-type and mutant exons were cloned into a pCAS2.1 vector (Gaildrat et al., 2010) and transiently transfected into HEK293T cells. Total RNA was extracted to evaluate alternatively spliced products by RT-PCR. Examining the change in the MaxEntScan score for the 11 genes revealed mutations in *ARID2*, *BAP1*, *BCOR*, *CDH1*, *PARP1*, *RAD51C*, *PTEN*, and *TSC2* having dramatically stronger splice scores in the presence of the mutation, while mutations in *BRCA1*, *KMT2A*, and *TP53* did not (Figure 5D). Except for *PTEN*, variants with stronger splice scores showed higher levels of the alternatively spliced product in the mini-gene assay when compared to the wild-type. Variants with moderate changes in splice score still showed evidence of alternatively spliced transcripts, revealing the importance of utilizing functional assays to evaluate the effect of mutations in a splicing context in addition to *in silico* predictions. The minigene assay confirmed 91% (10/11 genes) splicing alterations in all tested genes and sequencing confirmed the alternatively spliced products (Figure 5E; STAR Methods), suggesting a strong concordance between MiSplice predicted SCMs and the functional assay.

### Neoantigens Introduced by SCMs

**Figure 6:**
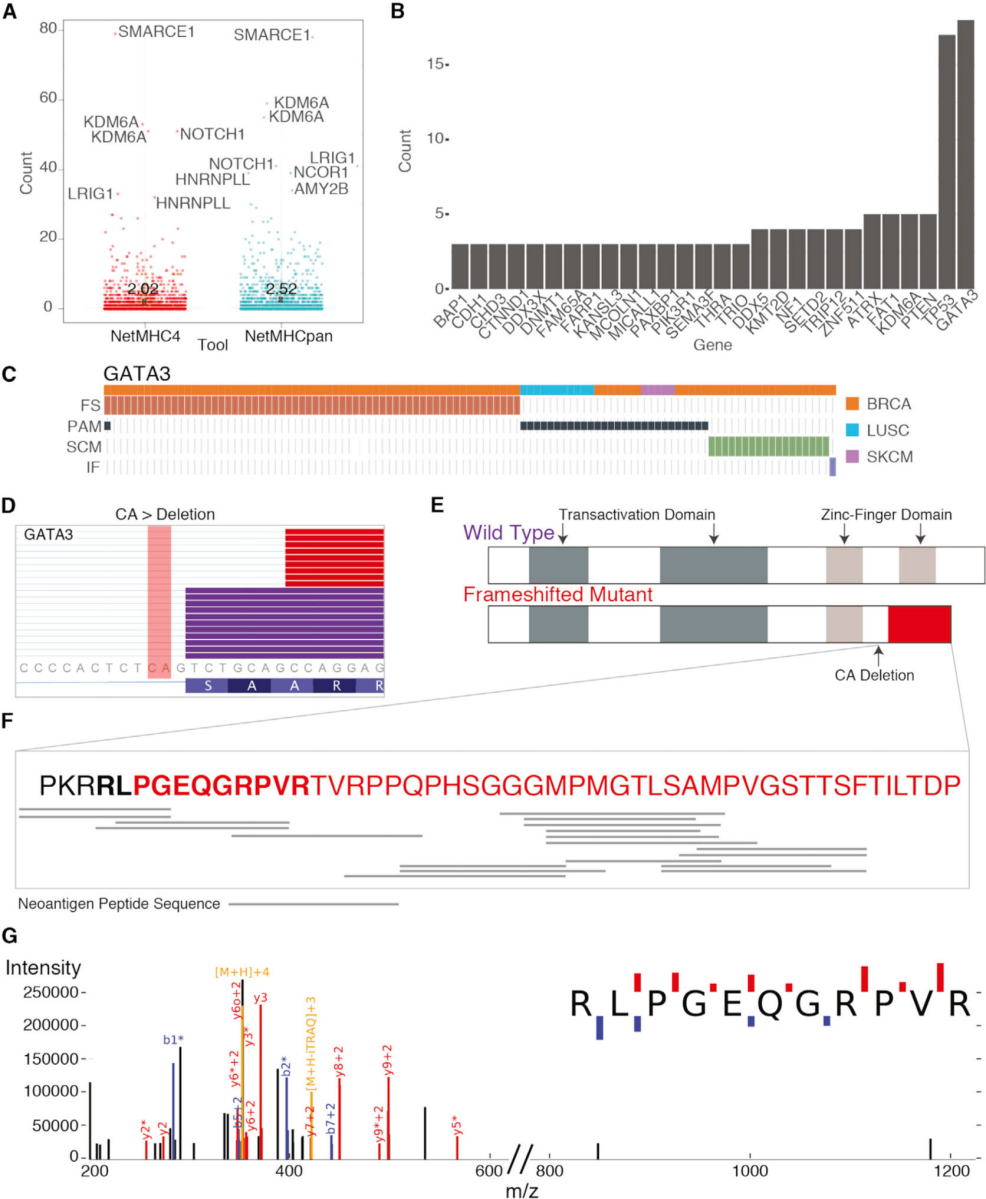
Schematic of GATA3 Splice-Site-Creating Mutations and Neoantigen Predictions (A) Distribution of neoantigens predicted by NetMHCpan and NetMHC4. Genes with the highest number of neoantigens labeled. Mean value for each tool indicated by X and labeled. (B) Genes with the largest recurrence of predicted neoantigens across the dataset. *GATA3* shows the highest recurrence. (C) Mutual exclusivity of protein-affecting mutation (PAM), frameshifting indel (FS), in-frame indel (IF), and splice-site-creating mutations (SCM) in *GATA3*. (D) IGV screenshot of *GATA3* splice-site-creating mutation, which disrupts the canonical splice site and utilizes a cryptic splice site 7 bp downstream. Mutant reads highlighted in red, and normal reads are in purple. CA deletion indicated in the figure. (E) Predicted functional domains disrupted because of the recurrent splice-site-creating mutation in *GATA3*. (F) Predicted neoantigen peptide sequences mapped to the frameshifted protein product for samples with *GATA3* SCMs. (G) Mass spectrum of GATA3 peptide in TCGA-AR-A1AP.

We have further investigated neoantigens produced by SCMs. By using the RefSeq transcript database, a total of 2,993 protein sequences were translated for transcripts containing mutation-induced alternative splice forms (Table S3). In the translation, we allowed for different transcripts from each SCM. The HLA types for each sample were adopted from the TCGA pancan immune working group (Synapse ID: syn5974636). NetMHC4 and NetMHCpan-3.0 (Andreatta and Nielsen, 2016) were used to predict the binding affinity between epitopes and the major histocompatibility complex (MHC) and showed a high concordance in total predicted neoantigens (Pearson = 0.94; Figure S2). We found that alternative splice forms for some important genes related to tumorigenesis, including *SMARC1*, *KDM6A*, and *NOTCH1*, are highly immunogenic and can contain 40 or more unique neoantigens (Figure 6A) (Dalgliesh et al., 2010; Papadakis et al., 2015). In addition, the mean number of neoantigens across SCMs from NetMHCpan-4.0 and NetMHCpan-3.0 are 2.0 and 2.5, respectively, which are both higher than the average number of around 1 for non-synonymous mutations. Furthermore, 28 genes contain recurrent neoantigen events (≥3) across samples (Figure 6B). In particular, *GATA3* has the highest recurrence and *GATA3* SCMs were mutually exclusive with other mutation types (Figure 6C). The CA deletion at chr8:8111433 disrupts the canonical splice site and an alternative splice site is used for creating the alternative splice form, which results in a frame shifted protein product spanning the Zinc-finger domain (Figures 6D and 6E). 19 unique neoantigen peptide sequences were mapped to the frameshifted protein product for the 16 samples (Figure 6F). We were further able to validate one alternative peptide sequence using mass spectrometry data from a recent proteogenomics study on 77 TCGA breast cancer patients (Mertins et al., 2016). For one sample with the highly recurrent and expressed *GATA3* SCM, we used MSGF+ to search publicly available mass spectrometry data for evidence of alternative GATA3 peptides. Figure 6G shows one identified mass spectrum supporting one alternative *GATA3* peptide, which covers two immunogenic peptides (KPKRRLPG and LIKPKRRLPG) predicted in TCGA-AR-A1AP.

**Figure 7:**
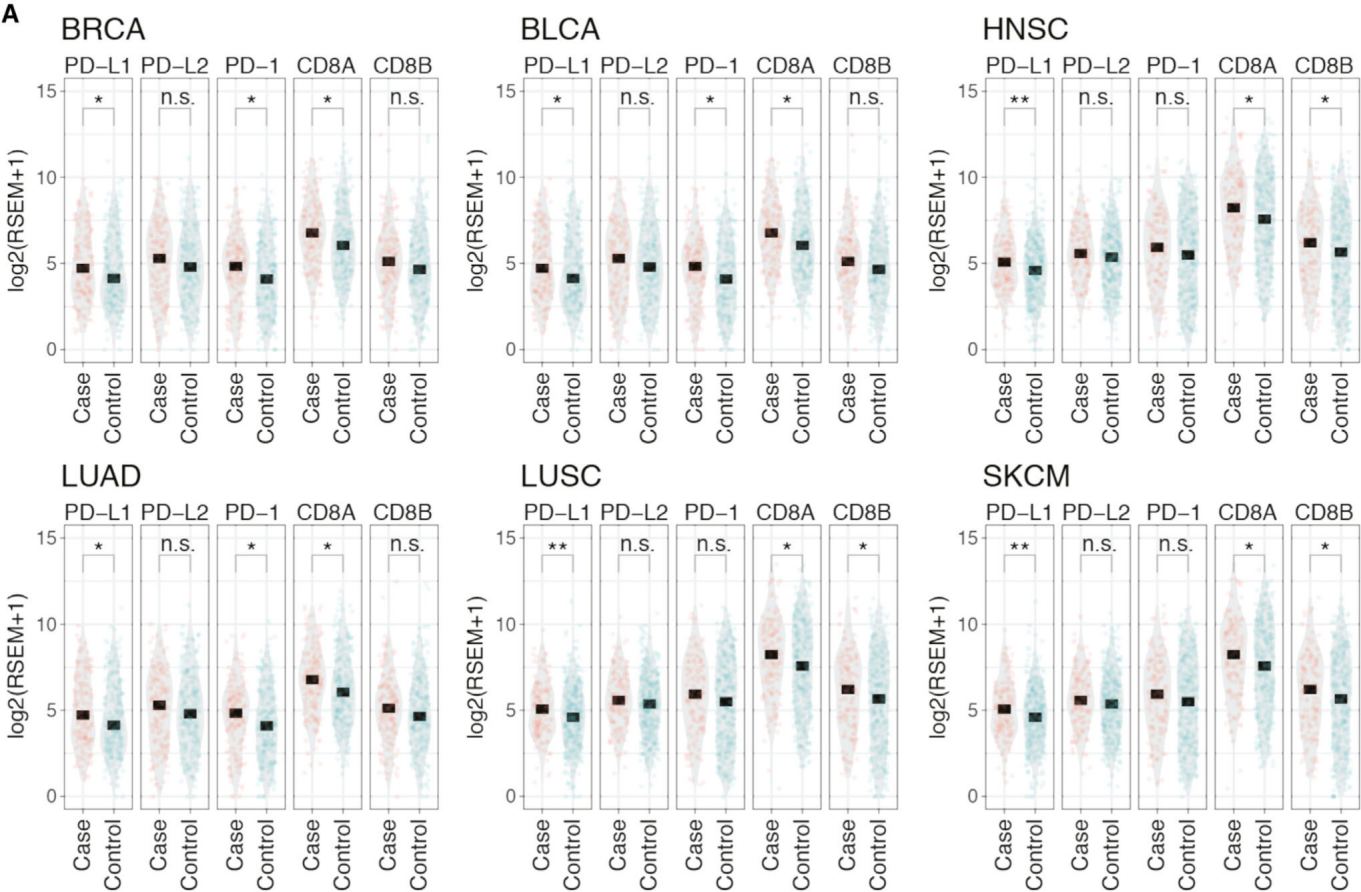
PD-L1, PD-L2, PD-1, CD8A, and CD8B Expression (A) Expression comparison of PD-L1, PD-L2, and T cell markers PD-1, CD8A, and CD8B between samples with (case) and without (control) SCMs across six cancer types. p values: * less than 0.05; ** < 0.01; and *** < 0.001; ns, not significant.

High neoantigen burden is associated with an elevated immune response (Turajlic et al., 2017). To test whether SCMs affect immune response, we compared the expression of T cell markers PD-1, CD8A and CD8B and PD1 immune checkpoint blockades PD-L1 and PD-L2 (Figure 7). We selected six cancer types (BRCA, BLCA, HNSC, LUAD, LUSC, and SKCM) with sufficient samples containing SCMs for adequate statistical power. Both T cell markers (PD-1, CD8A, and CD8B) and immune checkpoint blockade PD-L1 show increased expression in samples with SCMs compared to samples without SCMs (Figure 7), indicating alternative splice forms induced by SCMs increase the overall immunogenicity of these cancers. The highly expressed PD-L1 suggests PD-L1 immunotherapy as potential treatments for samples containing SCMs.

## DISCUSSION

In this study, we applied our newly developed bioinformatics tool called MiSplice (mutation-induced splicing) to systematically analyze splice-site-creating events that arise from somatic mutations. Our analysis shows MiSplice reliably identifies SCMs across multiple cancer types. Existing studies have largely focused on splice-disrupting events in known splice sites, but the current study substantially extends our knowledge into the realm of SCMs in human cancer. For instance, we found 1,016 splice site mutations not only disrupt the canonical splice site but also create an alternative splice site. We also found that hundreds of mutations that would traditionally be classified as missense, silent, indel, and nonsense are really acting as SCMs. Many important cancer-related genes harbor these mutations, such as *TP53*, *ATRX*, *BAP1*, *CTNNB1*, *RB1*, etc. It is noteworthy that we found five SCMs in *ATRX* among 288 LGG cases, likely leading to the disruption of ATRX function. A previous study has shown that loss of wild-type *ATRX* is associated with tumor growth in glioma (Koschmann et al., 2016).

Characterization of these alternative splice events show that most SCMs have a higher splice score, as measured by MaxEntScan, in the post-mutation alternative splice site as compared to the reference. These results are consistent with the preferential selection of these alternative sites as new splicing forms. For the splice-site mutation, the splice score associated with the canonical junction is coincidently decreased after mutation. However, while there is no difference in splice scores of canonical junctions before and after missense and silent mutations, the alternative splice site was often strengthened after mutation. This suggests silent and missense mutations instead act as modifiers of splicing by creating or strengthening cryptic sites within the exon as opposed to disrupting the canonical splice site. In addition, we found a significant enrichment of mutations at the −3 position in the 3′ splice site, the two dominant sequence contexts being aGag and agGag, where G is at the −3 position.

In cases in which the mutation is retained in the alternative splice junction, we distinguish mutations with two further categories, splice-in and splice-out. For splice-in mutations, we can characterize the association between mutations and cryptic splicing forms. For example, we found high concordance for RNA-seq reads supporting alternatively spliced junctions and mutations, suggesting the association between mutations and cryptic splicing forms.

The current study has greatly extended insights into the transcriptional ramifications of genomic alterations by identifying nearly 1,964 alternative splice sites introduced by somatic mutations and functionally validating 10 of 11 variants in a mini-gene splicing assay. These events were conventionally annotated as missense, silent, splice site, nonsense, or other mutations when, in fact, we have shown that they often create cryptic splice sites. This relative abundance of the alternative and wild-type product suggests varying levels of junction usage, depending on the context of the mutation, and emphasizes the importance of validating predictions using a functional assay to understand the full biological consequence. The alternative products may be therapeutically targetable in some cancer patients. For example, targeting neoantigens shows promising results in treating melanoma patients (Carreno et al., 2015). By further evaluating human leukocyte antigen (HLA) genotypes and binding affinities to the MHC, it is likely that new neoantigens from cryptic splice sites may be discovered. The current study reveals that alternative splice forms induced by SCMs are highly immunogenic and correlated with a high T cell immune response and an elevated PD-L1 expression, suggesting the potential for immunotherapy in these samples. Further investigation of the cryptic splice sites by mass spectra or target assay are needed to prioritize therapeutic targets in clinical trials.

## STAR★METHODS

Detailed methods are provided in the online version of this paper and include the following:

- KEY RESOURCES TABLE
- CONTACT FOR REAGENT AND RESOURCE SHARING
- EXPERIMENTAL MODEL AND SUBJECT DETAILS
- METHOD DETAILS
  ◦ Dataset Description
  ◦ MiSplice Pipeline
  ◦ Splice Site Score Estimation
  ◦ Neoantigen Prediction
  ◦ Manual Review
  ◦ Code Availability
  ◦ Mini-gene Splicing Assay
  ◦ Cell Culture
- QUANTIFICATION AND STATISTICAL ANALYSES

## STAR★METHODS

### CONTACT FOR REAGENT AND RESOURCE SHARING

Further information and requests for resources and reagents should be directed to and will be fulfilled by the Lead Contact, Li Ding (lding@wustl.edu).

### EXPERIMENTAL MODEL AND SUBJECT DETAILS

The Cancer Genome Atlas (TCGA) collected both tumor and non-tumor biospecimens from 10,224 human samples (https://cancergenome.nih.gov/abouttcga/policies/informedconsent). Here, we use variants from a publicly available mutation annotation file (MAF) complied by the MC3 working group (syn7824274).

### METHOD DETAILS

#### Dataset Description

Aligned RNA-seq bam files were analyzed using the ISB google. These cancer types are Acute Myeloid Leukemia [LAML], Adrenocortical carcinoma [ACC], Bladder Urothelial Carcinoma [BLCA], Brain Lower Grade Glioma [LGG], Breast invasive carcinoma [BRCA], Cervical squamous cell carcinoma and endocervical adenocarcinoma [CESC], Cholangiocarcinoma [CHOL], Colon adenocarcinoma [COAD], Esophageal carcinoma [ESCA], Glioblastoma multiforme [GBM], Head and Neck squamous cell carcinoma [HNSC], Kidney Chromophobe [KICH], Kidney renal clear cell carcinoma [KIRC], Kidney renal papillary cell carcinoma [KIRP], Liver hepatocellular carcinoma [LIHC], Lung adenocarcinoma [LUAD], Lung squamous cell carcinoma [LUSC], Lymphoid Neoplasm Diffuse Large B cell Lymphoma [DLBC], Mesothelioma [MESO], Ovarian serous cystadenocarcinoma [OV], Pancreatic adenocarcinoma [PAAD], Pheochromocytoma and Paraganglioma [PCPG], Prostate adenocarcinoma [PRAD], Rectum adenocarcinoma [READ], Sarcoma [SARC], Skin Cutaneous Melanoma [SKCM], Stomach adenocarcinoma [STAD], Testicular Germ Cell Tumors [TGCT], Thymoma [THYM], Thyroid carcinoma [THCA], Uterine Carcinosarcoma [UCS], Uterine Corpus Endometrial Carcinoma [UCEC], Uveal Melanoma [UVM]

#### MiSplice Pipeline

The MiSplice pipeline was developed to detect mutation-induced splicing events from RNA-seq data. It is written in Perl and incorporates two standard tools, samtools and MaxEntScan. The pipeline is fully automated and can run multiple jobs in parallel on LSF cluster. It executes the following steps:

1. Splitting large maf file into multiple smaller files with less mutations (currently, the default setting is 200).
2. Discovering splicing junctions within 20bps of the mutation with at least 5 supporting reads with mapping quality Q20 and then filtering canonical junctions by using the Ensembl 37.75 database. We selected 20bp as a cut-off since it is the farthest distance from the splice junction in a splice region.
3. Computing the number of supporting reads of above cryptic splice sites for control samples without mutations (Table S1).
4. Calculating the splicing scores for the cryptic splice sites via MaxEntScan.
5. Reporting the depth of each cryptic splice site via Samtools.
6. Filtering cryptic sites which fall in HLA loci or less than 5% of reads at the genomic location supporting the alternative junction of interest.
7. Further filtering cryptic sites by comparing the supporting reads in control samples. The final reported cryptic sites must stand as top 5% for the number of supporting reads in the case (with mutation).

#### Splice Site Score Estimation

For each cryptic splice site and nearby canonical splice site, the corresponding nucleotide sequences were first extracted for both the mutant and reference sequences (9-mer and 23-mer for donor and acceptor, respectively). Their splice scores as potential donor or acceptor sites were then estimated using MaxEntScan.

#### Neoantigen Prediction

For each predicted SCM, we use a curated RefSeq transcript database (version 20130722) to obtain the translated protein sequences for transcript containing alternative splice forms induced by SCMs. Different length of epitopes (8-mer, 9-mer, 10-mer and 11-mer) are constructed from the translated protein sequence. We use NetMHC3pan (Nielsen and Andreatta, 2016) and NetMHC4 (Andreatta and Nielsen, 2016) to predict the binding affinity between epitopes and MHC. Epitopes with binding affinity ≤ 500nM which are also not present in the wild-type transcript are extracted from the following neoantigen analysis.

#### Manual Review

All splice-site-creating mutations were manually reviewed using the integrative genomics viewer (http://software.broadinstitute.org/software/igv/). Mutations were placed into one of three categories: Pass, Complex, and No Support. Mutations were classified as complex if more than one alternatively spliced product was observed for the mutated sample.

#### Code Availability

MiSplice is written in Perl and is freely available from GitHub at https://github.com/ding-lab/misplice under the GNU general public license. MiSplice uses several independent tools and packages, including SamTools and MaxEntScan, all of which are likewise freely available, but which must be obtained from their respective developers. The MiSplice documentation contains complete instructions for obtaining and linking these applications into MiSplice.

#### Mini-gene Splicing Assay

Exons of interest and approximately 150 bp of their flanking intron sequences were PCR amplified from HEK293T genomic DNA using primers carrying restriction enzyme sites for BamH1 and MluI. PCR products were cleaned up using NucleoSpin PCR Cleanup (Macherey-Nagel) or DNA Clean and Concentrator-5 Kit (Zymo Research) and digested with BamHI and MluI. The digested pCAS2.1 vector and PCR products were ligated using T4 DNA Ligase (NEB). Mutations were introduced via Q5 Site-Directed Mutagenesis (NEB). WT and MUT constructs were confirmed by sequencing of the insert region. The plasmids were transiently transfected into HEK293T cells using Lipofectamine 2000 (ThermoFisher Scientific). 24 hr post transfection, cDNA was synthesized using 2 to 3 ug of total RNA with the Superscript III First-Strand Synthesis System (ThermoFisher Scientific) and priming with Oligo(dT)20. Finally, cDNA was amplified using pCAS-KO1-(5′-TGACGTCGCCGCCCATCAC-3′) and pCAS-R (5′-ATTGGTTGTTGAGTTGGTTGTC-3′) and the alternative splicing patterns were evaluated on a 2.5% agarose gel with ethidium bromide. Qiaquick Gel Extraction Kit (QIAGEN) was used to purify bands for sequencing (Figures S3, S4, S5, and S6; Tables S5, S6, and S7).

#### Cell Culture

HEK293T cells were cultured in Dulbecco’s modified Eagle’s medium (DMEM) supplemented with fetal bovine serum (FBS) and penicillin streptomycin.

### QUANTIFICATION AND STATISTICAL ANALYSES

MiSplice assesses the significance of the number of reads supporting the predicted alternative splice junction by comparing to read counts from a control cohort. Specifically, a frequency distribution is constructed from the control cohort, from which threshold values for 5% and 95% tails on the left and right, respectively, are determined. A series of logic tests is then conducted to discern the best explanation of the data. Possible verdicts are low or high expression if the datum is outside the 5% or 95% thresholds, respectively, average expression if no thresholds are exceeded, or no expression in this tissue if the thresholds are zero.

## Supplementary Material

1

2

3

4

5

6

## Figures and Tables

**KEY RESOURCES TABLE:** 

REAGENT or RESOURCE	SOURCE	IDENTIFIER
Experimental Models: Cell Lines		
Human: HEK293T cells	ATCC	https://www.atcc.org/products/all/CRL-3216.aspx
Oligonucleotides		
Primers for cDNA amplification pCAS-KO1-(5′-TGACGTCGCCGCCCATCAC-3′) pCAS-R (5′-ATTGGTTGTTGAGTTGGTTGTC-3′)	This paper	N/A
Primers for Q5 mutagenesis and restriction enzyme primers for amplifying exons of interest see Table S6	This paper	N/A
Recombinant DNA		
Plasmid: pCAS2	Inserm Laboratory	N/A
Software and Algorithms		
MaxEntScan	Yeo and Burge, 2004	http://genes.mit.edu/burgelab/maxent/Xmaxentscan_scoreseq.html
Samtools	Li et al., 2009	http://samtools.sourceforge.net/
MiSplice	In preparation	https://github.com/ding-lab/misplice
Integrative Genomics Viewer	Robinson et al., 2011	http://software.broadinstitute.org/software/igv/
Chemicals, Peptides, and Recombinant Proteins		
Nucleospin PCR Cleanup	Macherey-Nagel	740609.10
DNA Clean and Concentrator-5 Kit	Zymo Research	D4003
BamHI	New England Biomedicine	R0136S
MluI	New England Biomedicine	R0198S
T4 DNA Ligase	New England Biomedicine	M0202S
Q5 Site Directed Mutagenesis	New England Biomedicine	E0554S
Lipofectamine 2000	Thermofisher Scientific	12566014
Superscript III First-Strand Synthesis System	Thermofisher Scientific	18080051
Qiaquick Gel Extraction Kit	QIAGEN	28704
Other		
Public MC3 MAF	In preparation	https://gdc.cancer.gov
MSGF+	N/A	https://www.ncbi.nlm.nih.gov/pubmed/?term=25358478
Mass Spectra Data from 77 TCGA Breast Cancer Patients	N/A	https://cptac-data-portal.georgetown.edu/cptac/s/S029

## Highlights

- MiSplice applied to PanCancer data identifies 1,964 splice-site-creating mutations
- 26% and 11% of SCMs had been previously mis-annotated as missense and silent mutations
- SCMs may be more immunogenic than are missense mutations
- A mini-gene functional assay validates 10 of 11 predicted SCMs
