# Supplementary material for: Systematic Analysis of Splice-Site-Creating Mutations in Cancer
Source: Cell Rep. Author manuscript; Available in PMC 2018 Jul 23. (PMC6055527; doi:10.1016/j.celrep.2018.03.052)
Supplement: 1 [file NIHMS958979-supplement-1.pdf]

Cell Reports, Volume *23*

Supplemental Information

Systematic Analysis of Splice-Site-Creating Mutations in Cancer

Reyka G. Jayasinghe, Song Cao, Qingsong Gao, Michael C. Wendl, Nam Sy Vo, Sheila M. Reynolds, Yanyan Zhao, Héctor Climente-González, Shengjie Chai, Fang Wang, Rajees Varghese, Mo Huang, Wen-Wei Liang, Matthew A. Wyczalkowski, Sohini Sengupta, Zhi Li, Samuel H. Payne, David Fenyö, Jeffrey H. Miner, Matthew J. Walter, The Cancer Genome Atlas Research Network, Benjamin Vincent, Eduardo Eyras, Ken Chen, Ilya Shmulevich, Feng Chen, and Li Ding

Gene	Sample	Position	Reference	Mutation	Coding Change	Amino Acid Change	Transcript
ARID2	TCGA-FS-A1Z3	12: 46243434	T	A	c.1787T>A	p.Val596Glu	ENST00000334344
TSC2	TCGA-EE-A17Y	16:2098705	C	A	c.89C>A	p.Ser30Tyr	ENST00000219476
CDH1	TCGA-GC-A3I6	16:68863653	C	G	c.2392C>G	p.Leu798Val	ENST00000219476
TP53	TCGA-FG-A60J	17:7577157	T	G	c.783-2A>C	p.X261_splice	ENST00000269305
RAD51C	TCGA-32-1982	17:56772380	A	G	c.234A>G	p.Thr78Thr	ENST00000337432
BCOR	TCGA-DM-A1HA	X:39922049	A	G	c.4123C>T	p.Arg1375Trp	ENST00000378444.4
BAP1	TCGA-B0-5107 and TCGA-CJ-4637	3: 52442512	T	C	c.233A>G	p.Asn78Ser	ENST00000460680
PARP1	TCGA-66-2791	1:226550831	G	A	c.2817C>T	p.Ser939Ser	ENST00000366794
BRCA1	TCGA-D6-6823	17:41256883	A	C	c.301+2T>G	p.X101_splice	ENST00000471181
PTEN	TCGA-06-2559	10:89692993	G	T	c.477G>T	p.Arg159Ser	ENST00000371953
KMT2A	TCGA-55-7994	11:118366474	G	T	c.5423G>T	p.Trp1808Leu	ENST00000534358

Supplemental Table 5: Mutation information for splice site-creating mutations validated in minigene assay. Related to Experimental Procedures.

Gene	Primer Type	Forward Primer	Reverse Primer
ARID2	Genomic Primer	ATAAGGATCCTGGAATTGTTTAAAACCCAGCGA	AGCTACGCGTGACTGAAAATCCCAAGTAGGAA
ARID2	Q5 Mutagenesis	CATATTCATGaGGTAGGAGTAAAACGGAG	TGCCTGCCCATTGCTACT
TSC2	Genomic Primer	ATTTGGATCCTGTCTCCCGGGCTTTCCTA	ATATACGCGTCAGGTCACCAAGATCCTGACT
TSC2	Q5 Mutagenesis	AATCCCAGGTaTGCAGAGGGTAAAC	TGGCCTCGGTGTTCCCAG
CDH1	Genomic Primer	ATTTGGATCCCCAGTGCTATTTGGGAGACTT	ATATACGCGTGCTCAGGCAAGCTGAAAACAT
CDH1	Q5 Mutagenesis	CCCCCGGTATgTTCCCCGCCC	ACACTCATGAGGGTTGGTGCAACG
TP53	Genomic Primer	ATTAGGATCCGGACCTCTTAACCTGTGGCT	ATATACGCGTAGGAAAGAGGCAAGGAAAGGTG
TP53	Q5 Mutagenesis	TATCCTGAGTcGTGGTAATCTAC	GGAAAAGAGAAGCAAGAG
RAD51C	Genomic Primer	ATTAGGATCCTGCATTTTTATGTTTCTCCACTCCT	ATATACGCGTTGGTTTCCTGACGATAGTACAAAAT
RAD51C	Q5 Mutagenesis	ATGCTGGTACgTCTGAGTCAC	ATCTTGGTTTATTTGTGAGAC
BCOR	Genomic Primer	ATTTGGATCCTATCTTTAAAAACTGAAGCCGCTC	ATATACGCGTCTCCTCTAGGACAGGGGAGTG
BCOR	Q5 Mutagenesis	GGAGTCCAGGtGGGGATTGCC	TGAGGGATCAAGTGTTTGGTTTTGC
BAP1	Genomic Primer	TTACGGATCCGAGGCTTATGCTTTGCTCTGC	ATTGACGCGTAGAGAGTGGACTCAGACACCC
BAP1 (N78S)	Q5 Mutagenesis	GATATTGTGAgTAACATGTTCTTTGC	ATCATCAATCACGGACGTATC
BAP1 (I76T) - Control	Q5 Mutagenesis	GATGATGATAcTGTGAATAACATG	AATCACGGACGTATCATC
BAP1 (V62V) - Control	Q5 Mutagenesis	CCGGCGAAAGGTTTCTACC	GACCGGCGCTCTTCGA
PARP1	Genomic Primer	AATTGGATCCGCTCCTACATGGGGCAACAG	GCGTACGCGTCAGAGTTACGCTACCTCATCAT
PARP1	Q5 Mutagenesis	CACATATCAGtAAGTTACCCAAG	AAGCGTGCTTCAGTTCATAC
BRCA1	Genomic Primer	ATTAGGATCCGGTTTTCTACTGTTGCTGCATCTT	ATATACGCGTGGTCTTATCACCACGTCATAGAA
BRCA1	Q5 Mutagenesis	GTTTGGAGTGgAAGTGTTGAATATC	CTGTGTCAAGCTGAAAAG
PTEN	Genomic Primer	ATTAGGATCCGCATTGAGAGTCCTGACGAA	TGTAACGCGTAAACCTGTTTTCCAGGGACTGA
PTEN	Q5 Mutagenesis	GGGAAGTAAGtACCAGAGACAAAAAG	CATAGAAATCTAGGGCCTC
KMT2A	Genomic Primer	ATTAGGATCCGCATTGAGAGTCCTGACGAA	TGTAACGCGTAAACCTGTTTTCCAGGGACTGA
KMT2A	Q5 Mutagenesis	TATGCTCAGTtGCAGGAGCGA	ATTATGGTCAAGTGAAGGTG

Supplemental Table 6: Primer information for genes validated in minigene assay. Related to Experimental Procedures.

Type	RT-PCR Sequence
pCAS2.1	TGACGTCGCCGCCCATCACGCCTCCAGGCTGACCCTGCTGACCCTCCTGCTGCTGCTGCTGGCTGG**GG**ATA<br>GAGCCTCCTCAAATCCAAATGCTACCAGCTCCAGCAGCCAAGATCCAGAGAGTTTGCAAGACAGAGGCGAAG<br>GGAAGGTCGCAACAACAGTTATCTCCAAGATGCTATTCGTTGAACCCATCCTGGAGGTTTCCAGCTTGCCGAC<br>AACCAACTCAACAACCAAT
BAP1 Wild Type	CCCTGTATATGGATTTATCTTCCTGTTCAAATGGATCGAAGAGCGCCGGTCCCGGCGAAAGGTCTCTACCTTGG<br>TGGATGATACGTCCGTGATTGATGATGATATTGTGAATAACATGTTCTTTGCCCACCAG
BAP1 Mutant	CCCTGTATATGGATTTATCTTCCTGTTCAAATGGATCGAAGAGCGCCGGTCCCGGCGAAAGGTCTCTACCTTGG<br>TGGATGATACGTCCGTGATTGATGATGATATT
TP53 Wild Type	CTCGCTTAGTGCTCCCTGGGGGCAGCTCGTGGTGAGGCTCCCCTTTCTTGCGGAGATTCTCTTCCTCTGTGC<br>GCCGGTCTCTCCCAGGACAGGCACAAACACGCACCTCAAAGCTGTTCCGTCCCAGTAGATTACCA
TP53 Mutant	CTCGCTTAGTGCTCCCTGGGGGCAGCTCGTGGTGAGGCTCCCCTTTCTTGCGGAGATTCTCTTCCTCTGTGC<br>GCCGGTCTCTCCCAGGACAGGCACAAACACGCACCTCAAAGCTGTTCCGTCCCAGTAGATTACCACGA
BCOR Wild Type	CTTGCCATCGGCATTCTCCACGTAGTATTCCCCTGTCAGTGGCAATCCCCGCCTGGACTCCTGAGGGATCAAG<br>TGTTTGGTTTTGCACAGTCTCTTCCCGGATGGCTTCTCGCTGTTGTCGGTGTATTTCTGCAGCAGGGAGGCAG<br>CCTGGCAATCCTCTTCTTCGTCTGCACACAGCACATCTGTCTTCTGGTTTTCTTTAATTTTCTGCTGTTTGGCAG<br>GCGGCCTGGAGGCTGGTGCGCAGCTTGGCTGAGCCTGCTTTTTGCCGCCTGCACTGGTGGATGAAAGACTC<br>TTCATGGGCGGAGAGCCGGAGAACACAGGCAAGC
BCOR Mutant	CTGGACTCCTGAGGGATCAAGTGTTTGGTTTTGCACAGTCTCTTCCCGGATGGCTTCTCGCTGTTGTCGGTGT<br>ATTTCTGCAGCAGGGAGGCAGCCTGGCAATCCTCTTCTTCGTCTGCACACAGCACATCTGTCTTCTGGTTTTCT<br>TTAATTTTCTGCTGTTTGGCAGGCGGCCTGGAGGCTGGTGCGCAGCTTGGCTGAGCCTGCTTTTTGCCGCCT<br>GCACTGGTGGATGAAAGACTCTTCATGGGCGGAGAGCCGGAGAACACAGGCAAGC
RAD51C Wild Type	AAGTTGGGATATCTAAAGCAGAAGCCTTAGAAACTCTGCAAATTATCAGAAGAGAATGTCTCACAAATAAACCAA<br>GATATGCTGGTACATCTGAGTCACACAAGAAGTGTACAGCACTGGAACTTCTTGAGCAGGAGCATACCCAGGG<br>CTTCATAATCACCTTCTGTTCAGCACTAGATGATATTCTTGGGGGTGGAGTGCCCTTAATGAAAACAACAGAAAT<br>TTGTGGTGCACCAGGTGTTGGAAAAACACAATTATG
RAD51C Mutant	AAGTTGGGATATCTAAAGCAGAAGCCTTAGAAACTCTGCAAATTATCAGAAGAGAATGTCTCACAAATAAACCAA<br>GATATGCTG
KMT2A Wild Type	CAGTGGGATGTTACCAAACGCAGTGCTTCCACCTTCACTTGACCATAATTATGCTCAGTGGCAGGAGCGAGAG<br>GAAAACAGCCACACTGAGCAGCCTCCTTTAATGAAGAAAATCATTCCAGCTCCCAAACCCAAAGGTCCTGGAG<br>AACCAGACTCACCAACTCCTCTGCATCCTCCTACACCACCAATTTTGA
KMT2A Mutant	TTGCAGGAGCGAGAGGAAAACAGCCACACTGAGCAGCCTCCTTTAATGAAGAAAATCATTCCAGCTCCCAAAC<br>CCAAAGGTCCTGGAGAACCAGACTCACCAACTCCTCTGCATCCTCCTACACCACCAATTTTGA
PARP1 Wild Type	CTTTGACACTGTGCTTGCCCTTGGGTAACTTGCTGATATGTGAAGCGTGCTTCAGTTCATAC
PARP1 Mutant	TGATATGTGAAGCGTGCTTCAGTTCATAC
BRCA1 Wild Type	ACTCCAAACCTGTGTCAAGCTGAAAAGCACAAATGATTTTCAATAGCTCTTCAACAAGTTGACTAAATCTCGTAC<br>TTTCTTGTAGGCTC
BRCA1 Mutant	CTGTGTCAAGCTGAAAAGCACAAATGATTTTCAATAGCTCTTCAACAAGTTGACTAAATCTCGTACTTTCTTGTA<br>GGCTC
ARID2 Wild Type	AACGGTCTTTCCAAATCATACAGTGAAGAGAGTGGAGGATTCCAGTAGCAATGGGCAGGCACATATTCATGTGG<br>TAGGAGTAAAACGGAGGGCTATACCACTTCCCATTCAGATGTACTATCAGCAGCAACCAGTTTCTACTTCTGTTG<br>TTCGTGTTGATTCTGTTCCTGATGTATCTCCTGCTCCTTCACCTGCAG
ARID2 Mutant	AACGGTCTTTCCAAATCATACAGTGAAGAGAGTGGAGGATTCCAGTAGCAATGGGCAGGCACATATTCATGAG
PTEN Wild Type	TTGCACAATATCCTTTTGAAGACCATAACCCACCACAGCTAGAACTTATCAAACCCTTTTGTGAAGATCTTGACC<br>AATGGCTAAGTGAAGATGACAATCATGTTGCAGCAATTCACTGTAAAGCTGGAAAGGGACGAACTGGTGTAATG<br>ATATGTGCATATTTATTACATCGGGGCAAATTTTTAAAGGCACAAGAGGCCCTAGATTTCTATGGGGAAGTAAGG<br>ACCAGAGACAAAAAG
PTEN Mutant	TTGCACAATATCCTTTTGAAGACCATAACCCACCACAGCTAGAACTTATCAAACCCTTTTGTGAAGATCTTGACC<br>AATGGCTAAGTGAAGATGACAATCATGTTGCAGCAATTCACTGTAAAGCTGGAAAGGGACGAACTGGTGTAATG<br>ATATGTGCATATTTATTACATCGGGGCAAATTTTTAAAGGCACAAGAGGCCCTAGATTTCTATGGGGAA
CDH1 Wild Type	GACTTTGACTTGAGCCAGCTGCACAGGGGCCTGGACGCTCGGCCTGAAGTGACTCGTAACGACGTTGCACCA<br>ACCCTCATGAGTGTCCCCCGGTATCTTCCCCGCCCTGCCAATCCCGATGAAATTGGAAATTTTATTGATGAA
CDH1 Mutant	GACTTTGACTTGAGCCAGCTGCACAGGGGCCTGGACGCTCGGCCTGAAGTGACTCGTAACGACGTTGCACCA<br>ACCCTCATGAGTGTCCCCCG
TSC2 Wild Type	AGGGGTTTTCTGGTGCGTCCTGGTCCACCATGGCCAAACCAACAAGCAAAGATTCAGGCTTGAAGGAGAAGT<br>TTAAGATTCTGTTGGGACTGGGAACACCGAGGCCAAATCCCAGGTCTGCAGAGGGTAAACAGACGGAGTTTAT<br>CATCACCGCGGAAATACTGAGA
TSC2 Mutant	AGGGGTTTTCTGGTGCGTCCTGGTCCACCATGGCCAAACCAACAAGCAAAGATTCAGGCTTGAAGGAGAAGT<br>TTAAGATTCTGTTGGGACTGGGAACACCGAGGCCAAATCCCAG

Supplemental Table 7: Predicted alternative and wild-type RT-PCR splice products from mini-gene splicing assay. Related to Experimental Procedures.

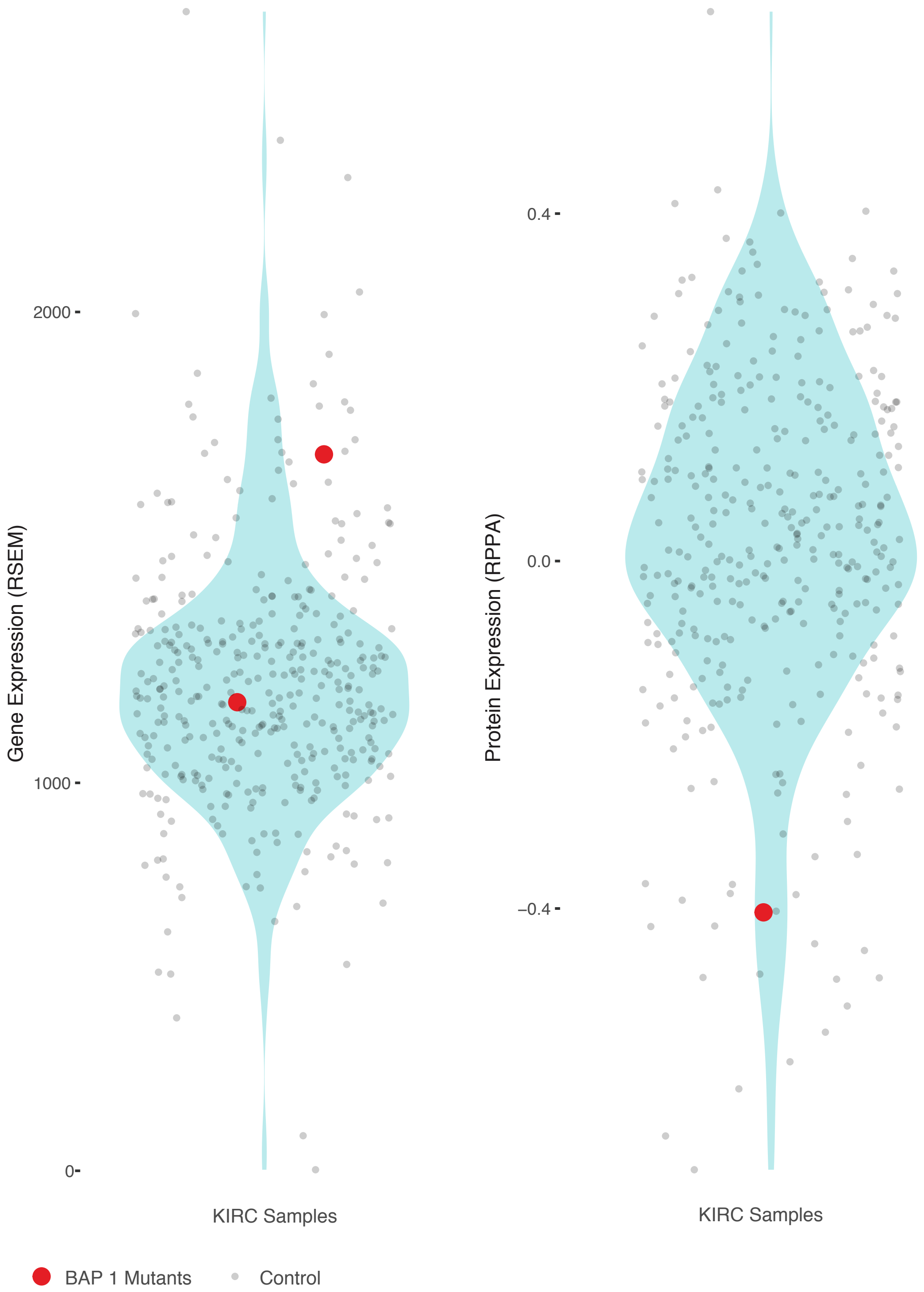

Figure S1. BAP1 gene and protein expression. Related to Figure 5. Violin plot of RSEM and RPPA data for control samples (grey) and novel splice creating mutant samples (red).

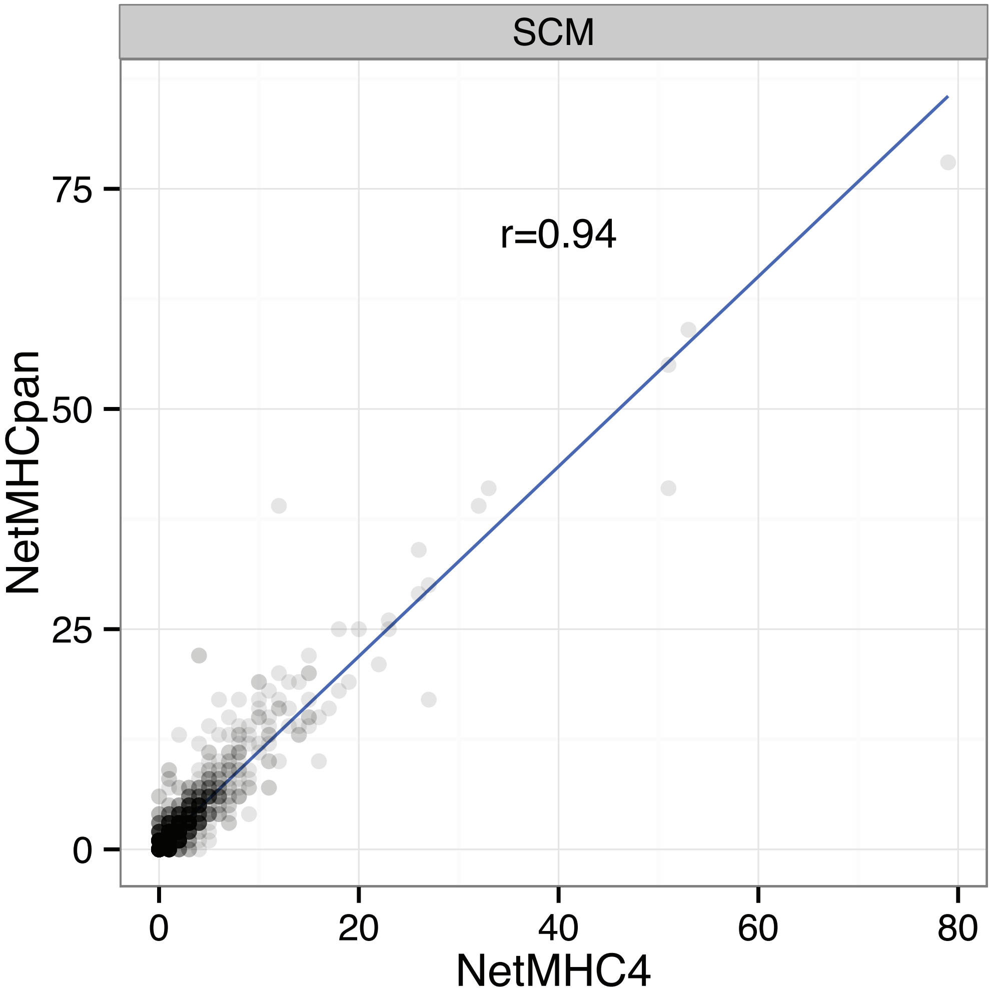

Figure S2. Comparison of neoantigen predictions betweeen NetMHC4 and NetMHCPan. Related to Figure 6. The predicted number of neoantigens from NetMHC4 and NetMHCpan-3.0

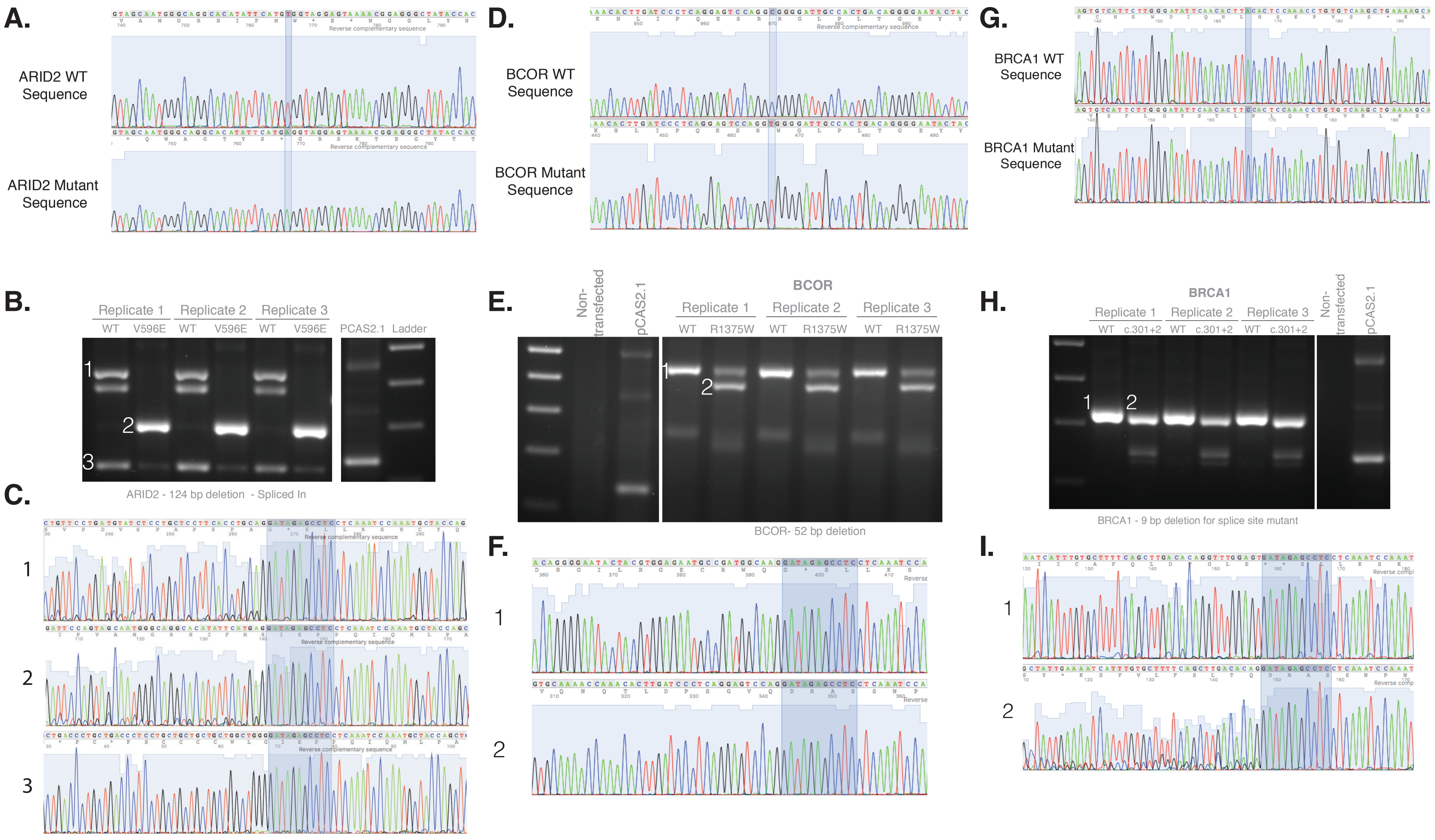

Figure S3. ***ARID2, BCOR, BRCA1*** **Mini-Gene results. Related to Figure 5.** (A,D,G) DNA chromatograms verifying *ARID2, BCOR, and BRCA1* wildtype and mutant sequencing results, respectively. Mutation position is highlighted. (B,E,H) Reverse transcriptase PCR (RT-PCR) with wild type and mutant plasmids, results in triplicate. Numbered bands are sequenced for confirmation. (C,F,I) DNA chromatograms of RT-PCR bands sequenced. Highlighted sequence indicates boundary of pCAS2.1 plasmid.

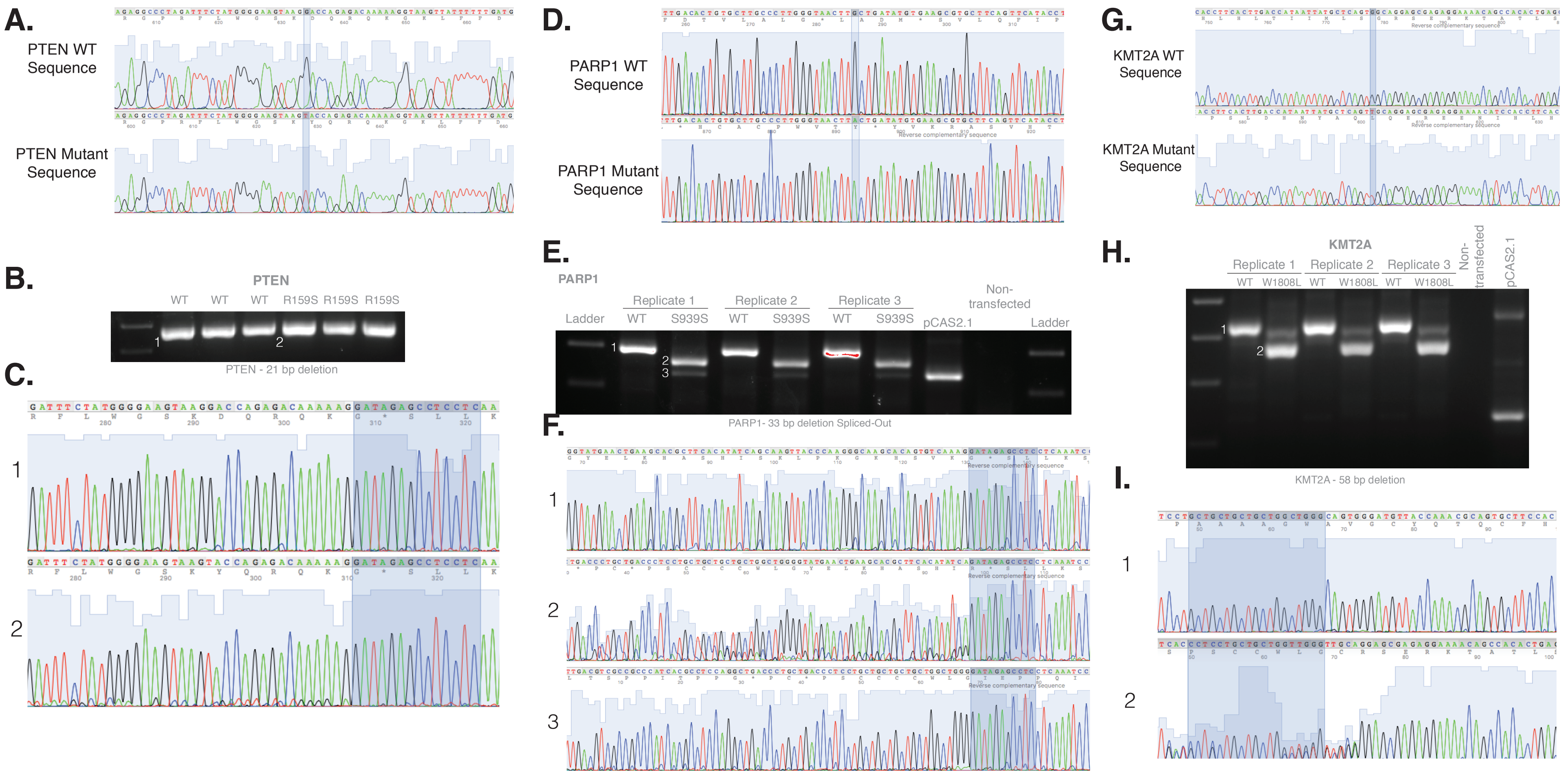

Figure S4. ***PTEN, PARP1, KMT2A*** **Mini-Gene results. Related to Figure 5.** (A,D,G) DNA chromatograms verifying *PTEN, PARP1, and KMT2A* wildtype and mutant sequencing results, respectively. Mutation position is highlighted. (B,E,H) Reverse transcriptase PCR (RT-PCR) with wild type and mutant plasmids, results in triplicate. Numbered bands are sequenced for confirmation. (C,F,I) DNA chromatograms of RT-PCR bands sequenced. Highlighted sequence indicates boundary of pCAS2.1 plasmid.

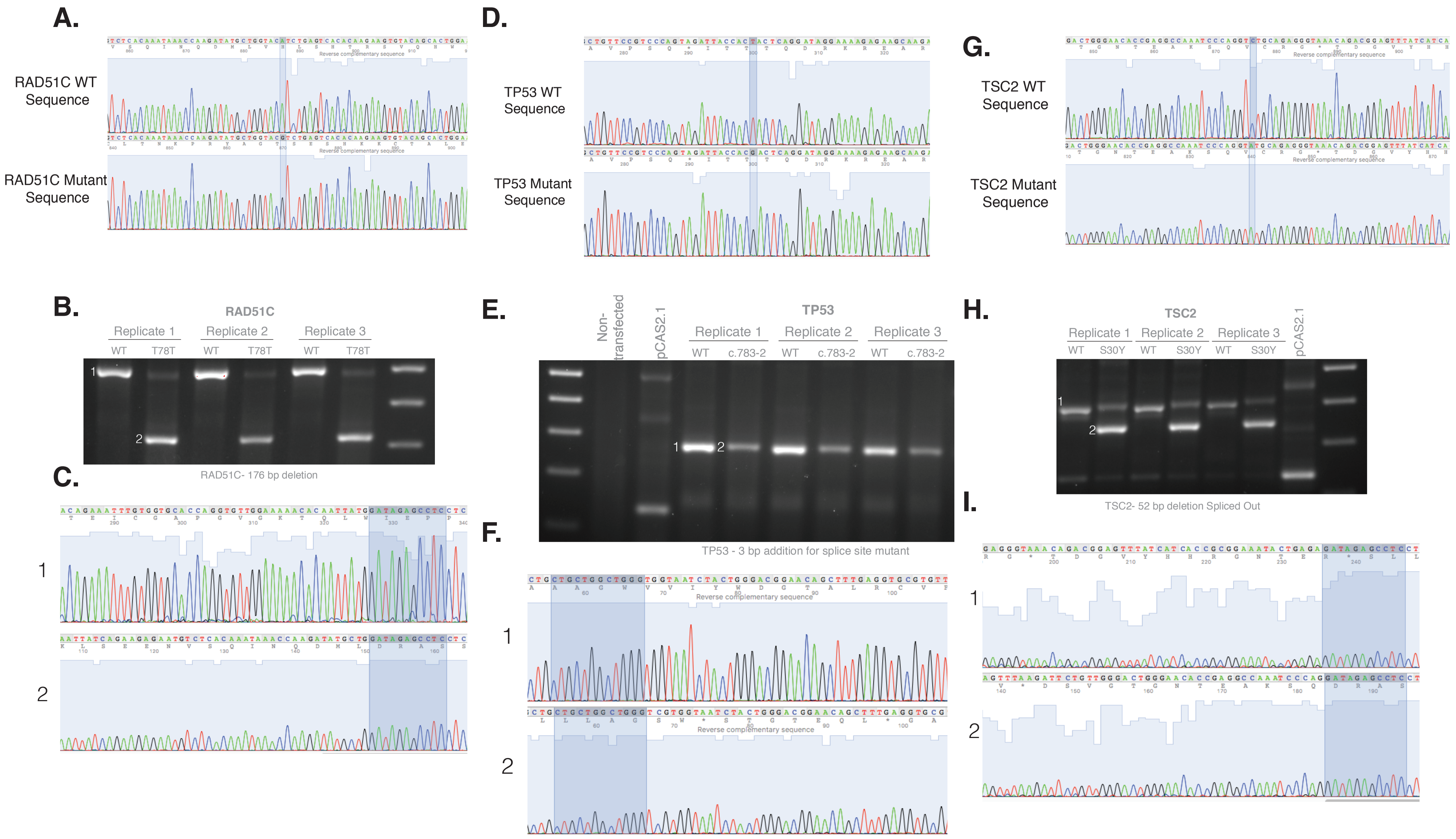

Figure S5. ***RAD51C, TP53, TSC2*** **Mini-Gene results. Related to Figure 5.** (A,D,G) DNA chromatograms verifying *RAD51C, TP53, and TSC2* wildtype and mutant sequencing results, respectively. Mutation position is highlighted. (B,E,H) Reverse transcriptase PCR (RT-PCR) with wild type and mutant plasmids, results in triplicate. Numbered bands are sequenced for confirmation. (C,F,I) DNA chromatograms of RT-PCR bands sequenced. Highlighted sequence indicates boundary of pCAS2.1 plasmid.

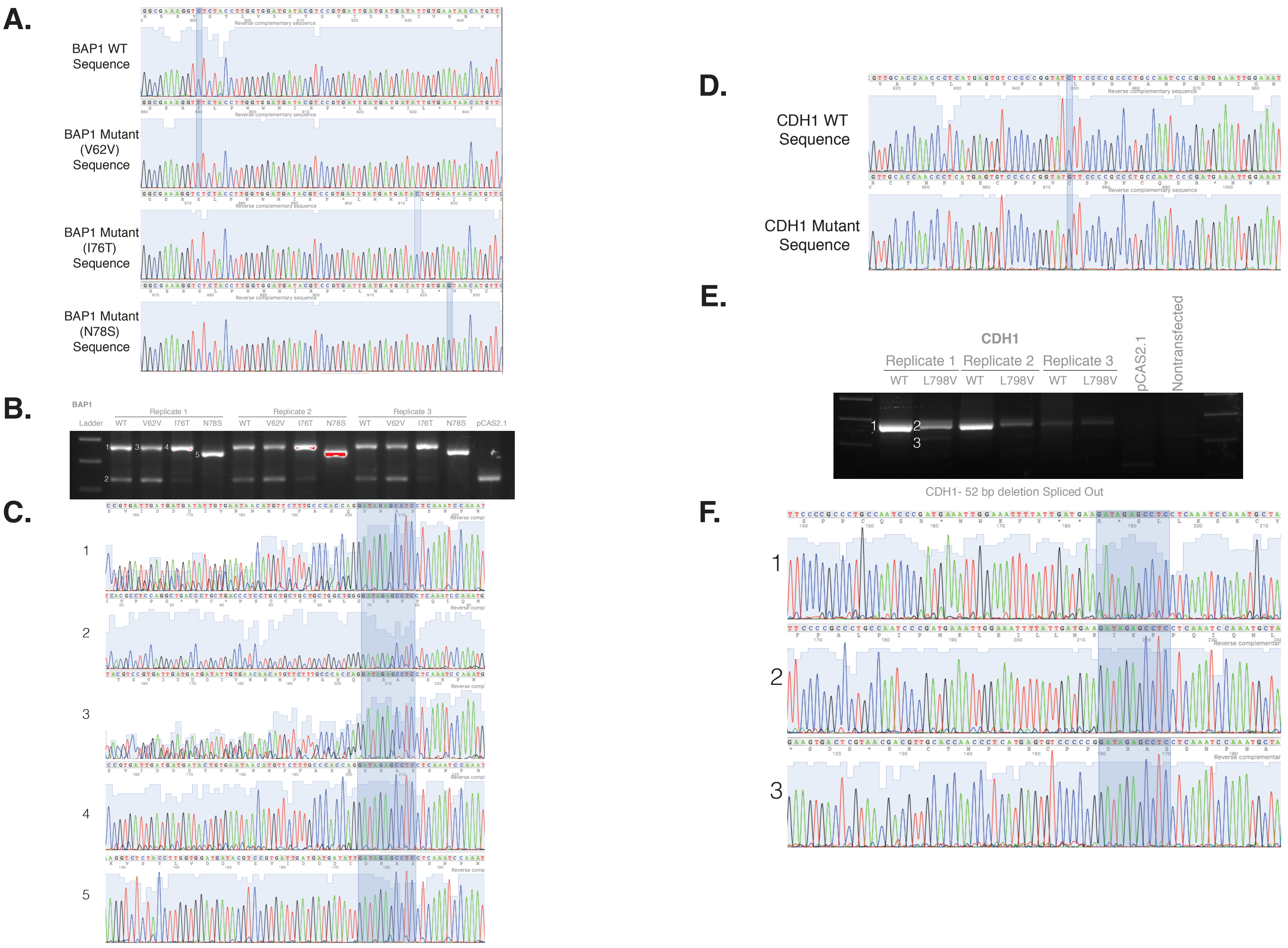

Figure S6. ***BAP1 and CDH1*** **Mini-Gene results. Related to Figure 5.** (A,D) DNA chromatograms verifying *BAP1* and *CDH1* wildtype and mutant sequencing results, respectively. Mutation position is highlighted. (B,E) Reverse transcriptase PCR (RT-PCR) with wild type and mutant plasmids, results in triplicate. Numbered bands are sequenced for confirmation. (C,F) DNA chromatograms of RT-PCR bands sequenced. Highlighted sequence indicates boundary of pCAS2.1 plasmid.
